# Miconazole-like Scaffold is a Promising Lead for *Naegleria fowleri*-Specific CYP51 Inhibitors

**DOI:** 10.1021/acs.jmedchem.3c01898

**Published:** 2023-12-12

**Authors:** Vandna Sharma, Valentina Noemi Madia, Valeria Tudino, Jennifer V. Nguyen, Anjan Debnath, Antonella Messore, Davide Ialongo, Elisa Patacchini, Irene Palenca, Silvia Basili Franzin, Luisa Seguella, Giuseppe Esposito, Rita Petrucci, Paola Di Matteo, Martina Bortolami, Francesco Saccoliti, Roberto Di Santo, Luigi Scipione, Roberta Costi, Larissa M. Podust

**Affiliations:** † Skaggs School of Pharmacy and Pharmaceutical Sciences, Center for Discovery and Innovation in Parasitic Diseases, University of California San Diego, La Jolla, California 92093, United States; ‡ Dipartimento di Chimica e Tecnologie del Farmaco, Istituto Pasteur-Fondazione Cenci Bolognetti, “Sapienza” Università di Roma, p.le Aldo Moro 5, Rome I-00185, Italy; § Dipartimento di Biotecnologie, Università degli Studi di Siena, Chimica e Farmacia via Aldo Moro 2, Siena 53100, Italy; ∥ Department of Physiology and Pharmacology “V. Erspamer”, “Sapienza″ Università di Roma, p.le Aldo Moro 5, Rome I-00185, Italy; ⊥ Dipartimento di Scienze di Base e Applicate per l’Ingegneria, “Sapienza” Università di Roma, Via Castro Laurenziano 7, Rome 00161, Italy; # D3 PharmaChemistry, Italian Institute of Technology, Via Morego 30, Genova 16163, Italy

## Abstract

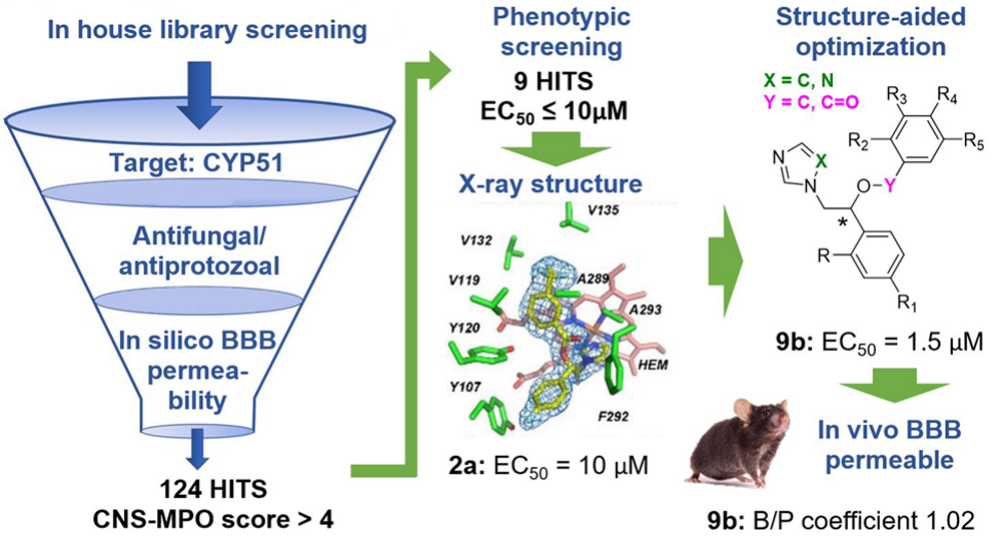

Developing drugs for brain infection by *Naegleria fowleri* is an unmet medical need. We used a combination of cheminformatics, target-, and phenotypic-based drug discovery methods to identify inhibitors that target an essential *N. fowleri* enzyme, sterol 14-demethylase (NfCYP51). A total of 124 compounds preselected *in silico* were tested against *N. fowleri*. Nine primary hits with EC_50_ ≤ 10 μM were phenotypically identified. Cocrystallization with NfCYP51 focused attention on one primary hit, miconazole-like compound **2a**. The *S*-enantiomer of **2a** produced a 1.74 Å cocrystal structure. A set of analogues was then synthesized and evaluated to confirm the superiority of the *S*-configuration over the *R*-configuration and the advantage of an ether linkage over an ester linkage. The two compounds, *S*-**8b** and *S*-**9b**, had an improved EC_50_ and *K*_D_ compared to **2a**. Importantly, both were readily taken up into the brain. The brain-to-plasma distribution coefficient of *S*-**9b** was 1.02 ± 0.12, suggesting further evaluation as a lead for primary amoebic meningoencephalitis.

## Introduction

The free-living amoeboflagellate, *Naegleria fowleri*, is commonly found in bodies of natural water (lakes and rivers) and in swimming pools with inadequate levels of chlorine. In the environment, *N. fowleri* occurs in three forms—a cyst (dormant form), a trophozoite (ameboid), and a flagellate. *N. fowleri* trophozoites feed mostly on bacteria but can also act as opportunistic pathogens, causing infection of the central nervous system (CNS) of animals including humans. Primary amoebic meningoencephalitis (PAM) due to *N. fowleri* is a fulminating brain infection that can result in death within days. PAM has a worldwide distribution, although it occurs most frequently in warmer regions and during hot summer months. It most commonly infects healthy children and young adults with recent recreational fresh water exposure.^1−4^ In the US, *N. fowleri* infection is considered “rare” with zero to eight cases reported annually but is likely under-reported.^5^ Most infected individuals die due to the rapid onset and destructive nature of the disease as well as to the lack of effective treatments.^6^ PAM cases often go unnoticed in countries with warm climates, poor health infrastructure, and ritual ablution practices that are common in certain religious groups.^7^ Based on the free-living amoeba registry maintained by the Centers for Disease Control and Prevention (CDC), the fatality rate of PAM is over 97%.^5^

Currently, there is no standard regimen for the treatment of *Naegleria* infection in humans. Only seven patients out of 381 reported PAM cases worldwide have been treated successfully with Amphotericin B (AmpB), either alone or in combination with other drugs.^8−12^ The CDC-recommended treatment for patients suspected of PAM includes combination therapy, administered intravenously, intrathecally, or orally, consisting of antimycotic drugs AmpB and fluconazole, antibiotics azithromycin and rifampin, an investigational agent miltefosine, and an anti-inflammatory drug dexamethasone. All the documented survivors of PAM received AmpB, but clinical use of AmpB is limited due to its toxicity, including acute infusion-related reactions and dose-related nephrotoxicity.^4^ Combination of AmpB with the antileishmaniasis agent, miltefosine, has shown promise, but not all patients who received miltefosine as part of their treatment regimens survived.^13^ Therefore, development of efficacious and safe drugs for PAM treatment remains an unmet medical need.

We^14−18^ and others^19−23^ have explored the steroidogenic pathway in free-living amoebae and have pharmacologically validated several steroidogenic enzymes as drug targets. Inhibition of sterol 14-demethylase (CYP51) with a variety of FDA-approved CYP51 inhibitors (conazoles) induced massive autophagocytosis in cultured *N. fowleri*, leading to cell death after 24 h of drug exposure.^15^ The amoebicidal effect of CYP51 inhibitors is due to inhibition of 14-demethylation of the endogenous CYP51 substrate in *N. fowleri*, 31-norlanosterol. 14-Demethylation of 31-norlanosterol is a prerequisite for subsequent 4α-demethylation and 24-methylation steps, leading to biosynthesis of essential ergosterol and ergosterol-like sterols.^14^ Depletion of ergosterol, concomitant with the accumulation of intermediates and end-products incompatible with normal permeability and fluidity of the *Naegleria* cell membrane, leads to the altered morphology and death of *N. fowleri* cells.^14,15^

**Chart 1:**
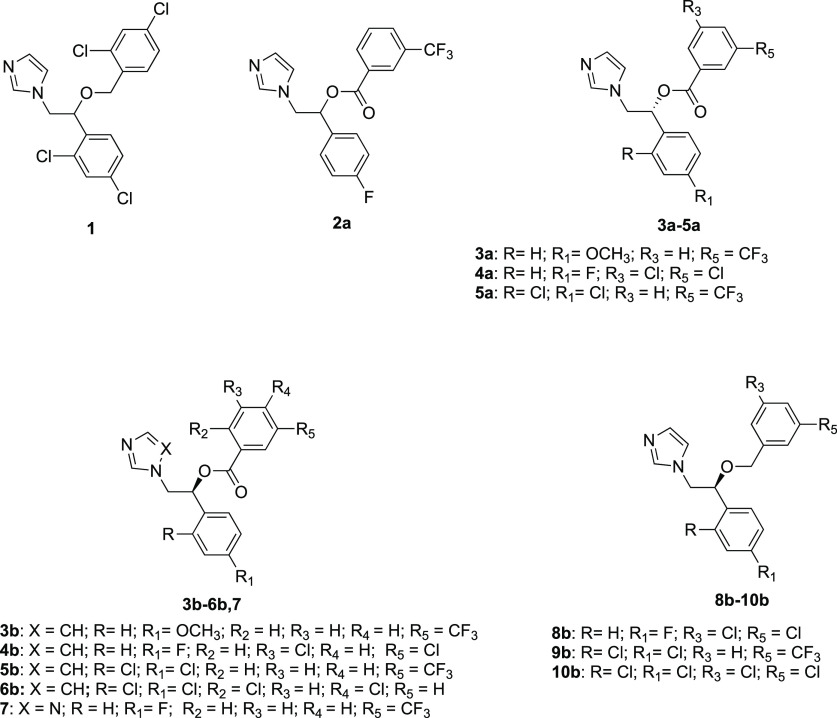
Structures of Miconazole (**1**) and the Azole Derivatives **2a**, **3a**–**5a**, **3b–6b**, **7**, and **8b**–**10b**

Conazoles have been used in combination with AmpB for the treatment of PAM patients. The first case of PAM survival in the United States involved a 9 year-old girl who was treated with both AmpB and miconazole in 1978 (**1**, Chart 1). These drugs were administered intravenously and intrathecally, in addition to oral rifampin, intravenous dexamethasone, and oral phenytoin.^24^ In more recent cases, miconazole was replaced with fluconazole, which is preferred when systemic treatment is required. This is because of the improved safety and predictable absorption of fluconazole when administered orally.^25^ In contrast to fluconazole, miconazole is approved for topical administration only in humans. It is used in the treatment of fungal or yeast infections of the skin or vagina and for the treatment of oropharyngeal candidiasis in patients 16 years and older. Nonetheless, there is a growing interest in miconazole due to its antiinflammatory^26^ and neuroprotective effects, which include remyelination of neural progenitor cells in models of multiple sclerosis,^27,28^ protection of brain blood vessels from rupture in a hemorrhagic stroke model,^29^ and ameliorating memory deficits in mice.^30^

We have previously reported that the anti-*Naegleria* activity of conazole analogues increases with an increase in molecular weight and hydrophobicity, while the CNS permeability decreases in the same order.^15^ Fluconazole, while having the lowest activity among the conazoles against *N. fowleri* European KUL strain (∼14 μM), is known to rapidly distribute through body tissues, including CNS compartments where it achieves concentrations greater than the MIC_90_ of common fungal pathogens.^31,32^ Posaconazole and itraconazole (EC_50_ of ≤10 nM) are superior in their amoebicidal effect to fluconazole and AmpB; however, they have poor blood–brain barrier (BBB) permeability. Slow accumulation in brain tissue upon repeated dosing^33−36^ may explain the lack of complete anti-*Naegleria* efficacy of posaconazole in a mouse model of PAM (two of six mice cured at a 20 mg/kg dose).^27^ Miconazole falls in between of these two extremes in terms of both anti-*Naegleria* potency (EC_50_ of <2 μM)^15^ and brain permeability.^37^ In this present work, the complementarity of the miconazole molecular scaffold to the *N. fowleri* CYP51 (NfCYP51) active site was demonstrated experimentally via a combination of cheminformatics, biochemistry, X-ray crystallography, and phenotypic cell-based methods. The analogues synthesized based on the miconazole template retained potency against the molecular target and were brain permeable in mice.

## Results and Discussion

### Design of Miconazole Analogues

A total of 124 compounds preselected *in silico* with an average MW of 345.6 ± 43.1 and cLogP of 3.79 ± 0.50 were tested against *N. fowleri* trophozoites. Nine hits were identified with EC_50_ ≤ 10 μM (**2a**–**2i**, see Table S1 in the Supporting Information). The top hit (**2a**, Chart 1) had a miconazole-like scaffold (**1**, Chart 1) and was singled out following cocrystallization with the NfCYP51 target. New analogues of this hit were then synthesized and characterized to assess (i) the stereo configuration at the chiral carbon center, (ii) the role of the ester linker, (iii) the optimal configuration of substituents at the phenyl and benzoyl moieties, and (iv) BBB permeability potential. By maintaining the azole ring, different substituents were introduced in the *o*- and/or *p*-position of the phenyl ring and in the *o*-, *m*-, and/or *p*-position of the benzoyl moiety. We introduced the following: (i) a trifluoromethyl group or two chlorine atoms on the benzoyl residue; (ii) a fluorine atom, a methoxy group, or two chlorine atoms on the aromatic ring of the phenyl moiety (Chart 1). To assess the role of the ester linker, we synthesized the ether counterparts by introducing (i) a trifluoromethyl group or two chlorine atoms on the benzoyl residue and (ii) a fluorine or two chlorine atoms on the aromatic ring of the phenyl moiety (Chart 1). The triazole analogue of hit compound **2a** was also synthesized. Notably, among the newly synthesized compounds, derivatives **6b** and **10b** were designed as the ester and 3,5-dichlorine-substituted miconazole analogues, respectively.

### Focused 124-Compound Library

The large, hydrophobic binding site of CYP51 favors molecules with physicochemical properties incompatible with BBB permeability.^38,39^ Thus, phenotypic and target-based screening alone may not yield quality hits for PAM. In the present work, we used the SwissADME cheminformatic tool^40^ to preselect compounds based on a combination of physicochemical parameters (lipophilicity, polar surface area, molecular weight, flexibility, hydrogen bond donor count, and most basic p*K*_a_), reflecting the compound’s CNS-multiparameter optimization (MPO) score.^41,42^ Starting from a library of 7000 compounds originally synthesized to target fungal CYP51,^39,43−46^ we identified a panel of 314 molecules endowed with antifungal and antiprotozoal activities. Among them, 124 compounds with an average molecular weight of 345.55 ± 43.13, cLogP of 3.79 ± 0.50, and topological polar surface area (TPSA) of 42.64 ± 15.85 were selected for the phenotypic screening against *N. fowleri*. All compounds contained an aromatic heterocycle (imidazole or 1,2,4-triazole) capable of coordinating the Fe center of the heme macrocycle in the NfCYP51 active site.

### Phenotypic Organism-Based Screen

The 124 selected compounds, all having a CNS-multiparameter optimization (MPO) score >4,^47^ were screened against the axenically cultured *N. fowleri* European KUL strain at 10 μM. Nine hits with ≥50% inhibition were identified (**2a**–**2i**, see Table S1 in the Supporting Information). An inhibition of 50% at 10 μM is comparable to the fluconazole reference potency (EC_50_ ∼ 14 μM) against the same strain.^15^

### Hit Cocrystallization with NfCYP51

**Figure 1:**
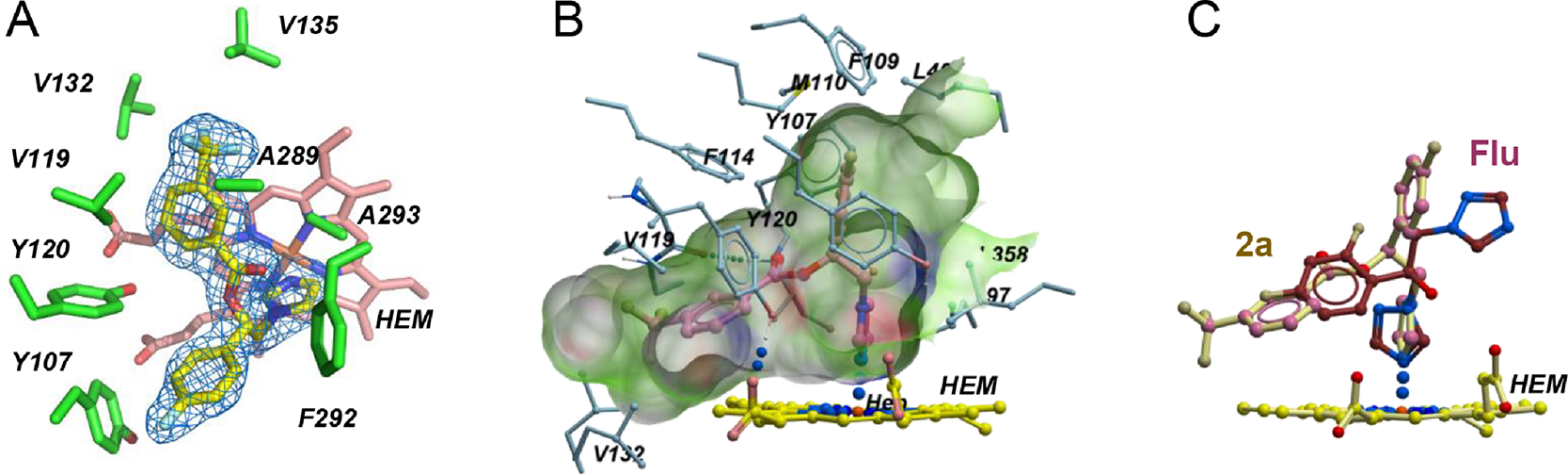
Drug–target interactions of **2a**. (A) Electron density map (blue mesh) at 1.75 Å delineates the binding pose of **2a** (pale yellow sticks). Amino acid residues within <4 Å of the inhibitor are shown in green sticks. Heme is pink. (B) Binding pocket (volume of 5178 Å^3^) accommodating **2a** (pink) is shown in shades of green. Amino acid side chains at the site boundaries are in blue sticks. (C) Superimposition of **2a** (pale yellow) and fluconazole (dark red, from PDB 6AY4) is shown. (B, C) Heme is yellow.

All nine hits were cocrystallized with recombinant NfCYP51. Only one compound, **2a** (used as a racemic mixture) (Chart 1), produced cocrystals with the target. The cocrystal structure of the NfCYP51–**2a** complex was determined to a resolution of 1.75 Å, which is comparable to the resolution of the NfCYP51–posaconazole complex (1.71 Å, PDB ID 5TL8) determined previously.^15^ For comparison, the NfCYP51–fluconazole complex diffracted only to a resolution of 2.7 Å (PDB ID 6AY4). This is consistent with the higher affinity of **2a** to NfCYP51, suggesting better target engagement. The well-defined electron density in the binding pocket adjacent to heme corresponds to the *S*-configuration at the chiral carbon center of **2a** (Figure 1A,B). Superimposition with fluconazole indicates that both molecules coordinate to the heme iron with the azole moiety, while other aromatic moieties of each compound are engaged in different sets of drug–target interactions (Figure 1C).

### **2a** Binding Pocket

**Figure 2:**
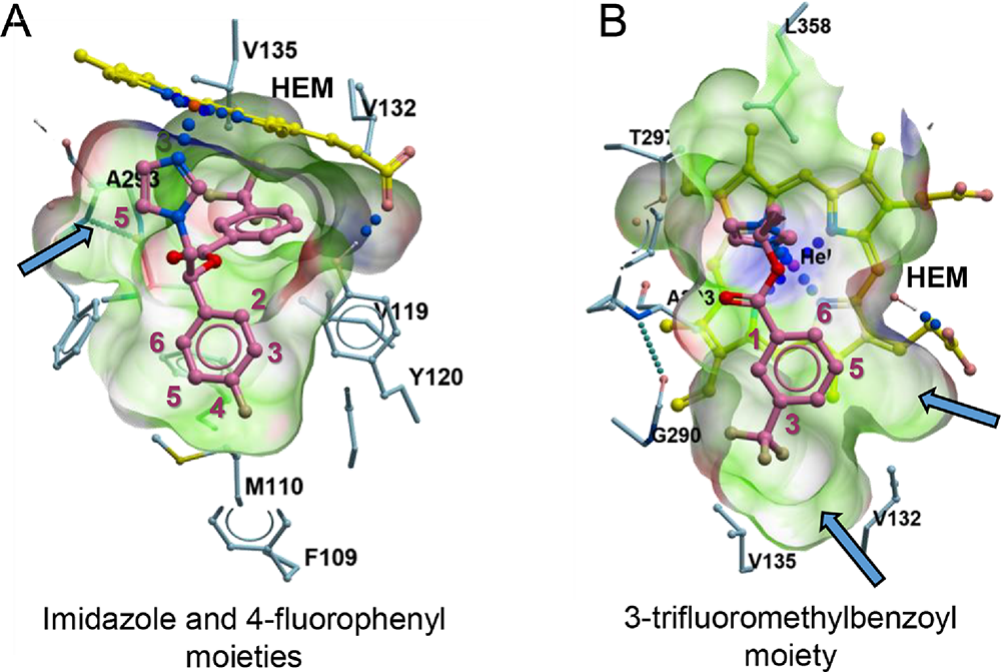
**2a** binding pocket. (A) Imidazole and 4-fluorophenyl moieties binding. (B) 3-Trifluoromethyl-benzoyl moiety binding. In (A) and (B), **2a** is in pink, heme (HEM) is in yellow, and amino acid residues at the pocket boundaries are in blue. Pocket boundaries are green. Blue arrows point at the adjacent void spaces.

The cocrystal structure of **2a** revealed three main groups of drug–target interactions (Figure 2). (a) Imidazole moiety. The aromatic nitrogen atom of the imidazole moiety provided a coordination bond to the heme iron (Figure 2A). There is a small cavity adjacent to C-5 of the imidazolyl moiety that may accommodate a small substituent. (b) The 3-trifluoromethyl-benzoyl moiety is somewhat coplanar to the heme plane (Figure 2B). Void spaces adjacent to the −CF_3_ group and C-5 suggest that halogens (i.e., chlorine and fluorine) or a variety of small alkyl groups with a gradual increase in the steric hindrance (such as methyl, ethyl, isopropyl, or *sec*-butyl groups) may improve drug–target fit and modulate physicochemical properties. (c) Finally, the 4-fluorophenyl moiety (Figure 2A) projects toward the long hydrophobic tunnel extending from the heme to the protein surface known to accommodate large hydrophobic moieties in higher molecular weight azole inhibitors (posaconazole and itraconazole).^15^ The C2–C3 edge of the 4-fluorophenyl moiety faces heme propionate (6.3 Å) and the OH-groups of Tyr107 (5.3 Å) and Tyr120. A void hydrophobic space at C-6 may accommodate another halogen substituent. For CNS compatibility, we propose staying within the binding envelope of **2a** and avoiding extending molecules into the hydrophobic tunnel.

**Figure 3:**
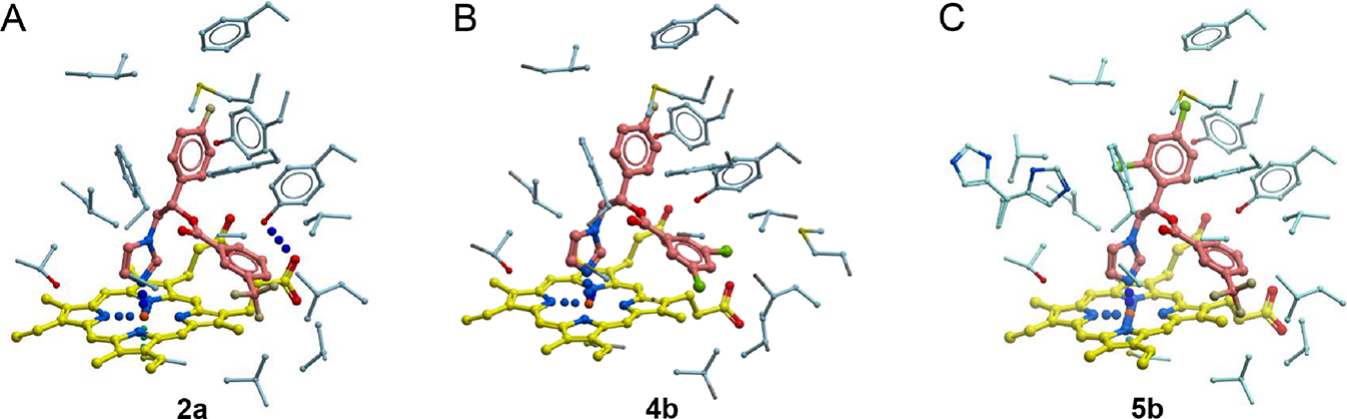
Binding mode of compounds (A) **2a**, (B) **4b**, and (C) **5b** to NfCYP51. In (A)–(C), the inhibitors are colored pink, heme is in yellow, and amino acid residues at the pocket boundaries are in blue. Heteroatoms are colored oxygen in red, nitrogen in blue, chlorine in green, and fluorine in olive.

Based on the NfCYP51–**2a** structure, we synthesized seven new analogues **3a**–**5a**, **3b**–**5b**, and **7** as pure enantiomers and evaluated them for binding affinity and anti-*N. fowleri* potency. The X-ray structures for the **4b** and **5b** drug–target complexes were determined at 1.81 and 2.10 Å, respectively (Table 1). Together with **2a**, these two structures demonstrated similar binding modes and built a foundation for a further hit-to-lead optimization strategy of the miconazole scaffold (Figure 3). Compounds **6b** (ester analog of miconazole) and **8b**–**10b** were synthesized to assess the contribution of the ester linker.

**Table 1:** Data Collection and Refinement Statistics

PDB ID	7RKR	7RKT	7RKW
inhibitor (PDB ID)	L49 (**2a**)	5UR (**5b**)	5TV (**4b**)
Data collection			
space group	*C*121	*C*121	*C*121
cell dimensions			
*a*, *b*, *c* (Å)	120.9, 55.4,71.5	121.7, 55.2,72.5	121.3, 55.3,72.1
α, β, γ (deg)	90.0, 100.1, 90.0	90.0, 100.2, 90.0	90.0, 100.1, 90.0
molecules in AU	1	1	1
wavelength	1.11587	1.11587	1.11587
resolution (Å)	1.76	2.10	1.81
*R*_sym_ or *R*_merge_ (%)	9.6 (451.4)^a^	9.2 (238.5)	5.6 (355.0)
*I*/σ*I*	8.94 (0.36)	9.02 (0.58)	12.19 (0.36)
completeness (%)	98.1 (77.7)	96.1 (65.6)	93.7 (58.7)
redundancy	6.4 (4.2)	6.3 (3.4)	6.1 (3.6)
Crystallization conditions			
	30 mM CaCl_2_; 4.50% v/v Jefframine M-600, pH 7.0; 33% v/v PEG MME^b^ 550; 100 mM Bis-Tris propane, pH 7.0	30 mM CaCl_2_; 4.55% v/v Jefframine M-600, pH 7.0; 33% v/v PEG MME 550; 100 mM Bis-Tris propane, pH 7.0	30 mM CaCl_2_; 3.18% v/v Jefframine M-600, pH 7.0; 33% v/v PEG MME 550; 100 mM Bis-Tris propane, pH 7.0
Refinement			
no. of reflections	43404	25517	38337
*R*_work_/*R*_free_ (%)	18.8/23.9 (53.8/54.9)	17.9/23.9 (48.6/56.1)	18.9/24.0 (67.7/65.3)
no. of atoms			
protein	3608	3628	3610
heme	43	43	43
inhibitor	27	28	25
solvent	99	38	63
Wilson plot *B* value	41.9	67.2	51.6
mean *B* value	48.2	79.6	60.9
*B*-factors			
protein	48.6	80.7	61.7
heme	34.4	58.2	43.1
inhibitor	36.6	67.2	47.1
solvent	49.6	74.1	58.1
R.m.s deviations			
bond lengths (Å)	0.017	0.013	0.017
bond angles (deg)	1.046	1.641	1.864

a Data for the highest resolution shell are shown in parentheses.

b PEG MME: polyethylene glycol monomethyl ether.

### Chemistry

**Scheme 1:**
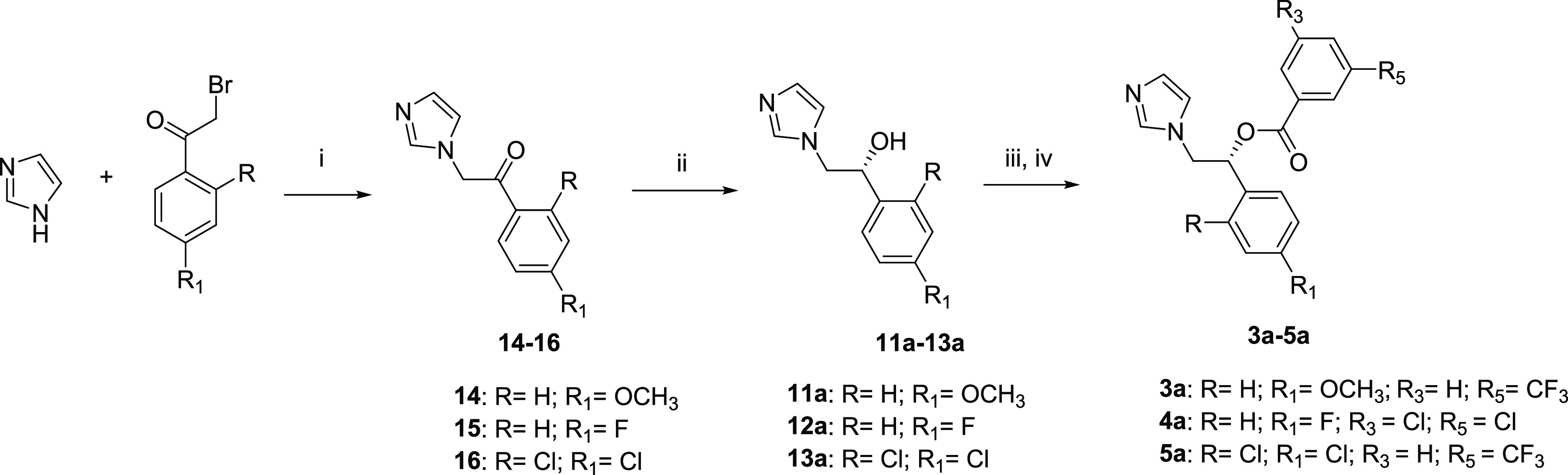
Synthetic Route to *R*-Enantiomers **3a**–**5a** Reagents and conditions: (i) DMF, 5–10 °C, 3 h, 48–57% yield; (ii) (*S,S*)-TsDPEN Ru-(*p*-cymene)Cl, DCM, Et_3_N, HCOOH, N_2_, room temp, 26 h, 31–88% yield; (iii) NaH, CH_3_CN, room temp, 2 h; (iv) proper benzoyl chloride, 24 h, reflux, 21–26% yield over two steps.

The synthesis of hit compounds **2a**–**2i** (see Table S1 in the Supporting Information) was carried out as previously reported.^44,45,48,49^ The *R*-enantiomers **3a**–**5a** were synthesized in accordance with the procedures outlined in Scheme 1. A mixture of the appropriate phenacyl bromide with 1*H*-imidazole in dimethylformamide (DMF) afforded the desired ketones **14**–**16**, which were reduced *via* catalytic asymmetric hydrogenation in the presence of a chiral diamine ligand complexed with ruthenium ((*S,S*)-TsDPEN Ru-(*p*-cymene)Cl) in dichloromethane (DCM), Et_3_N, and HCOOH, as described in the literature.^50,51^ The obtained *R*-configured alcohols **11a**–**13a** were treated with sodium hydride in anhydrous acetonitrile, and subsequently, the proper acyl chloride was added to give compounds **3a**–**5a**, as previously described.^43^

**Scheme 2:**
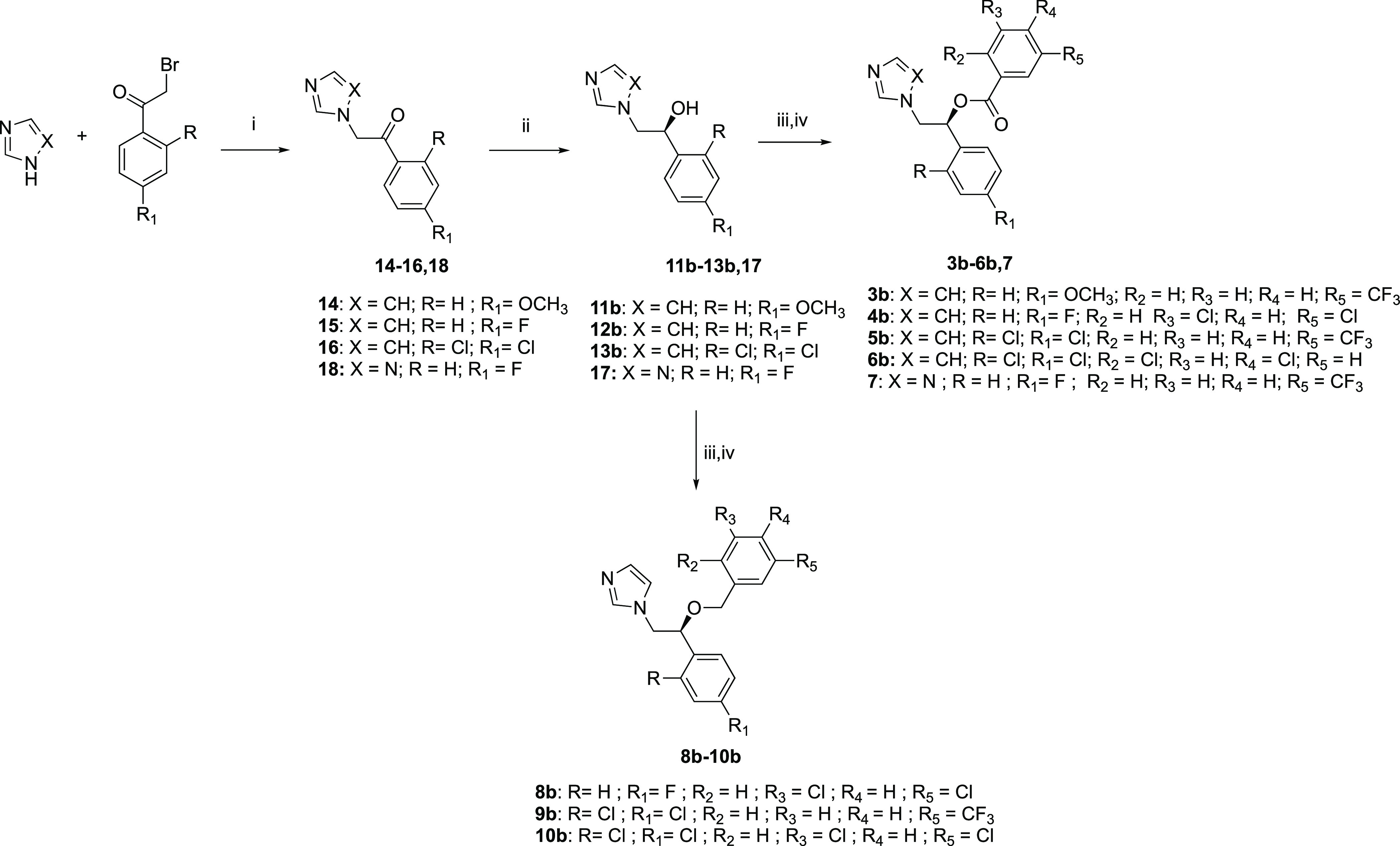
Synthetic Route to *S*-Enantiomers **3b**–**6b**, **7**, and **8b**–**10b** Reagents and conditions: (i) DMF, 5–10 °C, 3 h for **14**–**16**, acetone, TEA reflux, 8 h for **18**, 48–57% yield; (ii) (*R,R*)-TsDPEN Ru-(*p*-cymene)Cl, DCM, Et_3_N, HCOOH, room temp, 26 h, 33–58% yield; (iii) NaH, CH_3_CN, −5 °C to room temp (or room temp for **11b**–**13b**, **17**), 2 h; (iv) proper benzyl halide or proper benzoyl chloride (for compounds **10b**–**12b**, **16**), 24 h, 0 °C to room temperature (or reflux for compounds **11b**–**13b**, **17**), 6–44% yield over two steps.

The synthesis of *S*-enantiomers **3b**–**6b** and **7** was performed, as reported in Scheme 2. The synthetic approach resembles that described above for compounds **2a**–**4a**. Noteworthily, the synthetic pathway of compound **7** starts with the reaction of 2-bromo-1-(4-fluorophenyl)ethan-1-one (commercially available) with 1*H*-1,2,4-triazole in the presence of Et_3_N in acetone, yielding a ketone derivative, **18**. This compound underwent catalytic asymmetric hydrogenation^50^ in a similar fashion to that described in Scheme 1, giving the *S*-configured alcohol **17** that was acylated^43^ with 3-(trifluoromethyl)benzoyl chloride to furnish derivative **7**.

### Evaluation of Binding and Biological Activities of the Newly Synthesized Compounds

We assessed the anti-*Naegleria* potency, binding affinity to the NfCYP51 target, and cocrystallization propensity of the newly synthesized analogues **3a**–**5a**, **3b**–**6b**, **7**, and **8b**–**10b**. Compounds **4b** and **5b** were the only two new analogues that produced cocrystals with NfCYP51 (Figure 3). Two chlorine atoms introduced on the aromatic ring of the benzoyl moiety (**4b**) or phenyl moiety (**5b**) notably improved the *K*_D_ and EC_50_, compared to the parental hit, **2a**; however, the potency of both analogues was inferior to miconazole (Table 2). The methoxy-substituent was inferior to fluorine in the 4-fluorophenyl moiety (**3a** and **3b**). Overall, the EC_50_ correlated with *K*_D_ and cocrystallization propensity of the analogues and the *S*-configuration was superior to the *R*-configuration for all tested enantiomer pairs. The hydrolytically unstable ester moiety featured in **2a** and the first seven analogues distinguished them from miconazole.

**Table 2:**
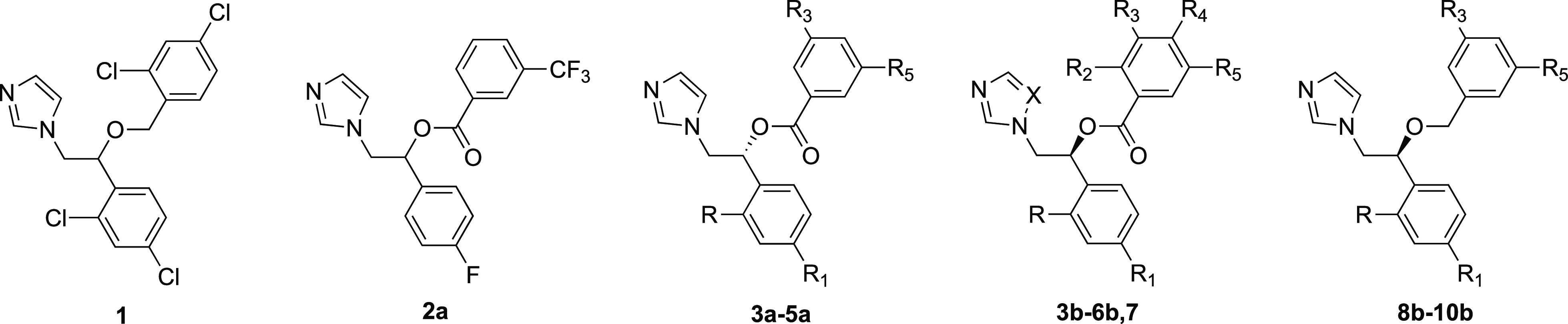
*In Vitro* Activities and Binding of the Reference Compounds Miconazole (**1**) and Fluconazole and of the Newly Synthesized Compounds **2a**, **3a**–**5a**, **3b**–**6b**, **7**, and **8b**–**10b**

Cpd	R	R_1_	R_2_	R_3_	R_4_	R_5_	X	stereo configuration	MW (g/mol)	EC_50_^a^ (μM)	*K*_D_^b^ (μM)	PDB ID
**2a**								mixed	379.33	∼50% inhibition at 10 μM	0.088 ± 0.004	7RKR
**3a**	H	OCH_3_		H		CF_3_		R	390.36	69% inhibition at 50 μM	N/D^c^	
**3b**	H	OCH_3_	H	H	H	CF_3_	CH	S	390.36	24.0 ± 0.9	N/D	
**4a**	H	F		Cl		Cl		R	379.21	21.50 ± 0.04	0.173 ± 0.006	
**4b**	H	F	H	Cl	H	Cl	CH	S	379.21	4.80 ± 0.05	0.028 ± 0.002	7RKW
**5a**	Cl	Cl		H		CF_3_		R	429.22	10.20 ± 0.03	0.161 ± 0.008	
**5b**	Cl	Cl	H	H	H	CF_3_	CH	S	429.22	4.30 ± 0.05	0.070 ± 0.002	7RKT
**6b**	Cl	Cl	Cl	H	Cl	H	CH	S	430.11	2.60 ± 0.03	≤0.005^d^	
**7**	H	F	H	H	H	CF_3_	N	S	379.31	20.60 ± 0.04	0.087 ± 0.010	
**8b**	H	F		Cl		Cl		S	365.23	2.40 ± 0.04	0.007 ± 0.002	
**9b**	Cl	Cl		H		CF_3_		S	415.24	1.50 ± 0.02	≤0.005	
**10b**	Cl	Cl		Cl		Cl		S	416.13	3.50 ± 0.01	≤0.005	
**MIC**^e^ (**1**)	Cl	Cl	Cl	H	Cl	H	CH	mixed	416.10	1.40 ± 0.02	≤0.005	
**FLU**^f^								N/A	306.30	13.9 ± 0.01^15^	0.141 ± 0.021	6AY4

a Compound concentration corresponding to 50% growth inhibition determined from dose–response curves: experiments performed in triplicate against axenically cultured *N. fowleri* trophozoits. Standard deviation for each compound was calculated from three independent experiments.

b *K*_D_ was determined at 0.5 μM NfCYP51; the inhibitor concentration was 0.025–0.5 μM. The standard deviation for each compound was calculated from three independent titrations.

c N/D = not determined.

d Estimated from linear dependence of binding from compound concentration.

e MIC = miconazole.

f FLU = fluconazole.

The hydrolytic instability of the ester moiety is consistent with the high clearance and short plasma half-life of **2a** observed in a single-dose PK experiment. **2a** administered intraperitoneally at 20 mg/kg could be detected at low levels, in both plasma (4 ng/mL) and brain (0.1 ng/mg), only 30 min post injection, while the hydrolysis product, 3-trifluoro benzoic acid, was one of the main circulating metabolites for up to 24 h. The X-ray structures for the **2a**, **4b**, and **5b** drug–target complexes showed that the ester moiety is not directly involved in the interactions with NfCYP51. Thus, another set of compounds, **8b**–**10b**, was synthesized, where the ester moiety was replaced with a hydrolytically more stable ether. Specifically, **8b** and **9b** were the ether counterparts of **4b** and **5b**, respectively, while **10b** was the 3,5-chlorine-substituted miconazole analogue. Indeed, in miconazole, two chlorine substituents are in the *para*/*ortho* configuration, while the *meta/meta* configuration was supported by the X-ray cocrystal structure of **2a** that predicted the potential interference of V132 with a *para*-substituent. Finally, miconazole analogues **6b** carrying the ester moiety were also synthesized.

**Figure 4:**
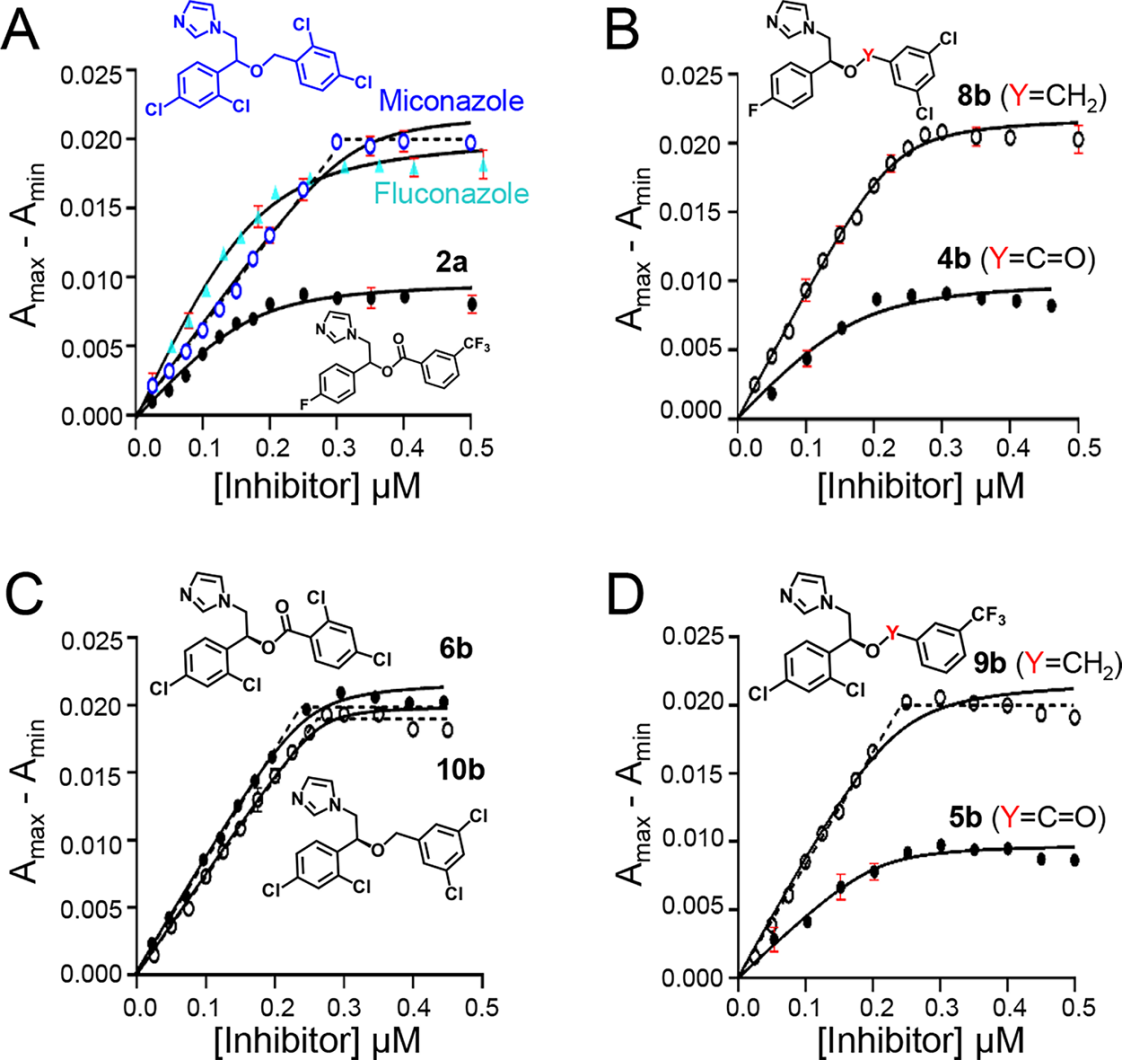
Compounds bind to NfCYP51. Binding isotherms of (A) miconazole (blue), fluconazole (cyan), and **2a** (black), (B) **4b** and **8b**, (C) **6b** and **10b**, and (D) **5b** and **9b**. Solid and hollow circles represent ester and ether derivatives, respectively. *A*_max_ is the absorbance at 430 nm, and *A*_min_ is the absorbance at 410 nm. The NfCYP51 concentration is 0.5 μM. Solid curves represent the Morrison binding equation fit, whereas dotted lines demonstrate the linear dependence of the data points on inhibitor concentrations. Binding experiments were performed in triplicate, and standard deviations in three independent titrations are shown in red; for most of the data points, deviations are smaller than the size of symbols and thus are invisible.

Most of the analogues from this latest set showed linear binding to NfCYP51 with an increase in compound concentration from 0.025 to 0.5 μM, until the target saturation is reached (Figure 4). Linear dependence does not allow for accurate calculation of *K*_D_. Reducing the target concentration below 0.5 μM is prohibited by the sensitivity of this UV–vis spectroscopy measurements using available instrumentation. We can only estimate *K*_D_ to be ≤5 nM based on the transformation of the parabolic isotherms into linear saturation curves that gradually occurs at target concentrations exceeding *K*_D_ by ∼100-fold. Compared to their ester counterparts, analogues **8b**–**10b** had an improved EC_50_, higher binding affinity (Table 2), and better target saturation (Figure 4). Against *N. fowleri*, **9b** was equipotent to miconazole, while **8b** and **10b** were slightly inferior. The higher binding affinity/target saturation and higher biological activity of the ether congeners are likely due to a better drug–target engagement achieved due to the higher flexibility of the ether linker. Also, an obvious advantage of the ether moiety over the ester moiety is its high plasma stability and the reported miconazole elimination half-life is 24 h.^52^

The binding curves plateau at ∼0.3 μM (instead of 0.5 μM). This observation indicates that not all binding sites participate in inhibitor binding. A plausible explanation of this phenomenon is irreversible deterioration of the heme Fe thiolate bond upon binding of azole inhibitors, as we demonstrated elsewhere by UV–vis spectroscopy for CYP51 of *N. fowleri* and *Acanthamoeba castellanii*.^53^ Depending on experimental conditions, deterioration rates are on a minute-to-hours time scale that is consistent with protein conformational motions. The broad Soret band indicates an enormous structural heterogeneity and flexibility of the heme pocket; close inspection of the spectra suggests that multiple species are present.^53^

### Blood–Brain Barrier Permeability in Mice

To experimentally evaluate brain penetration of the best acting compounds **8b** and **9b**, BBB permeability was determined *in vivo*. Compounds **8b** and **9b** were administered at 40 mg/kg i.p. dose, using miconazole (**1**) as a reference. Miconazole was used as a reference throughout this present work because among existing azole drugs, miconazole molecular scaffolds were singled out as the most complementary to the NfCYP51 active site. After 1 h of drug exposure, blood and brain samples were collected and analyzed by means of HPLC-ESI-MS/MS. As shown in Table 3, both **8b** and **9b** confirmed their capability of crossing the BBB in mice. **8b** was equally permeable to miconazole, with an average brain distribution coefficient (brain/plasma ratio) of 0.43 ± 0.08, while **9b** distributed to the brain with a B/P coefficient of 1.02 ± 0.12.

**Table 3:** Blood–Brain Barrier Permeability of the Lead Compounds^a^

	**miconazole (1)**		**8b**		**9b**	
mice	*M1*	*M2*	*M3*	*M4*	*M5*	*M6*
brain (μg/g)	1.332 ± 0.101	0.442 ± 0.036	1.005 ± 0.021	0.232 ± 0.011	0.034 ± 0.004	0.042 ± 0.002
plasma (μg/mL)	2.535 ± 0.091	1.027 ± 0.217	1.990 ± 0.101	0.656 ± 0.018	0.038 ± 0.008	0.037 ± 0.03
brain/plasma (B/P)	0.53	0.43	0.51	0.35	0.89	1.14

a Amounts of **1**, **8b**, and **9b** (40 mg/kg ip doses) quantified in duplicate in brain and blood. Reported as mean values ± standard deviation of triplicate analysis and calculated brain/blood ratio.

## Conclusions

Using a combination of cheminformatics, target-based, and phenotypic drug discovery methods, we have identified a lead scaffold suitable for further development of drug candidates to treat brain infection with *N. fowleri*. Only compounds with a CNS-multiparameter optimization (MPO) score >4 were selected for primary phenotypic screening against *N. fowleri* followed by cocrystallization with the molecular target, NfCYP51. We identified a promising hit, **2a**, which resembled miconazole, the first member of the “conazole” pedigree that in the 1970s was used in combination with AmpB and rifampin to treat PAM. It was later replaced with fluconazole because of improved safety and better absorption. Fluconazole remains the only CYP51 inhibitor in a combination of drugs recommended by the CDC for the treatment of PAM. In laboratory assays, fluconazole kills *N. fowleri* at concentrations much higher than other azole drugs. We reason that the success of an inhibitor in treating *N. fowleri* infection in humans would sum from complementarity to a molecular target combined with an adequate BBB permeability, allowing a drug to enter the central nervous system from systemic circulation.

Compound **2a** showed better potency and drug–target complementarity than fluconazole; the follow up ester analogues **4b** and **5b** retained the binding mode and improved the binding and potency. The hydrolytically more stable ether analogues, **8b** and **9b**, had further improved binding and drug potency; **9b** with a 3-trifluoromethyl substituent was equipotent to miconazole. The ester analogue of miconazole, **6b**, and the *meta*/*meta* chlorine-configured **10b** were comparable to miconazole in binding affinity and slightly inferior to miconazole in anti-*Naegleria* potency.

The *in vivo* assessment of the BBB permeability of active compounds, **8b** and **9b**, confirmed that both were detected in brain tissue. Plasma and brain concentrations of **8b** were similar to those of miconazole; **9b** demonstrated better brain permeability than miconazole and **8b**. The B/P distribution coefficient of ∼1 suggests free distribution of **9b** to the brain.

This study demonstrated for the first time complementarity between miconazole congeners and the NfCYP51 target and predicted medicinal chemistry modifications within the binding envelope that would retain potency yet gain BBB permeability. These results now warrant further development of miconazole analagos into anti-*Naegleria* drug candidates.

## Experimental Section

### Chemistry: General

Solvents and reagents were of analytical grade and, when necessary, purified and dried by standard methods. Merck silica-gel 60 F_254_ plates were used for analytical TLC. Column chromatography was performed on silica gel (Merck, 70–230 mesh). Melting points were determined with a Büchi 530 capillary apparatus and are uncorrected. Infrared (IR) spectra were recorded on a PerkinElmer Spectrum-one spectrophotometer. NMR spectra were recorded on a Bruker AC 400 spectrometer at 400 MHz for ^1^H and 100 MHz for ^13^C; the following abbreviations were used: s for singlet, bs for broad singlet, d for doublet, t for triplet, dd for double doublet, m for multiplet; chemical shifts are given in d with respect to the residual solvent signal; coupling constants are given in Hz. DMSO-*d*_6,_ CD_3_CN, acetone-*d*_6_, and CD_3_OD of 99.9% isotopic purity (Aldrich) were used. Mass spectra were recorded on a ThermoFinnigan LCQ Classic LC/MS/MS ion trap equipped with an ESI source and a syringe pump; samples (10^–4^–10^–5^ M in methanol (MeOH)/H_2_O 80:20) were infused in an electrospray system at a flow rate of 5–10 μL min^–1^; when necessary, 50 μL of 10^–2^ M HCOOH was added to the sample solutions to promote the analyte ionization; the ESI-MS data are given as *m*/*z*, with mass expressed in amu.

The enantiomeric excess (ee) of the (*R*)- and (*S*)-enantiomers was evaluated on 0.5 mg mL^–1^ compound samples (MeOH),^54^ by chiral HPLC, using a 150 mm × 4.6 mm i.d. Phenomenex LUX Cellulose 5 μm column (Phenomenex, Italy).^55,56^ The HPLC apparatus consisted of a Shimadzu HPLC system (Shimadzu Corporation, Kyoto, Japan), pump (LC-10AD), autosampler (SIL-10AD), UV detector (SPD-10A), column oven (CTO-10C), and system controller (SCL-10A) with a PC control program (LabSolution, LC Solution Version 1.21 SP1).

Compounds were eluted with MeOH/H_2_O + 0.2% Et_3_N (isocratic mode composition in the range 90:10 v/v to 87:13 v/v), at 1 mL min^–1^ flow rate, recording the chromatograms at 254 nm.

A total of 5–10 mg of each compound was synthesized for phenotypic organism-based screening and target-based evaluation assays. All compounds are >95% pure, as determined by combustion analysis. Analytical results agreed to within ±0.40% of the theoretical values.

## General Experimental Procedures

### General Procedure A (GP-A) to Obtain Ketones (**14**–**16** and **18**)

A mixture of the proper phenacyl bromide (4 mmol) and 1*H*-imidazole or 1*H*-1,2,4 triazole (20 mmol) in DMF (3 mL) was stirred at 5–10 °C for 3 h. This solution was poured into water, and the precipitate was filtered, washed with water, and dried on anhydrous Na_2_SO_4_. The crude product was purified by column chromatography on silica gel using ethyl acetate (EtOAc)/MeOH/Et_3_N (9:0.5:0.5 for compounds **14** and **15**) or EtOAc/MeOH (9:1 for compounds **16** and **18**) as an eluent. For each compound, phenacyl bromide, melting point (°C), yield (%), IR, ^1^H NMR, and elemental analysis are reported.

### General Procedure B (GP-B) to Obtain *R*-Configured Alcohols (**11a**–**13a**)

A nitrogen atmosphere was established in a three-neck flask containing the appropriate imidazolyl-ethanone (1 mmol) and [(*S,S*)-TsDPEN]Ru-(*p*-cymene)Cl (0.001 mmol) before the addition of dichloromethane (7 mL) and Et_3_N (5 mmol). Formic acid (5 mmol) was added over a period of an hour. The mixture was stirred at room temperature for 26 h. NaHCO_3_ saturated solution was added cautiously and the organic layer with water and brine and dried over anhydrous Na_2_SO_4._^47^ The solvent was removed under vacuum, affording the desired product. For each compound, imidazolyl-ethanone, melting point (°C), yield (%), IR, ^1^H NMR, and elemental analysis are reported.

### General Procedure C (GP-C) to Obtain *S*-Configured Alcohols (**11b**–**13b** and **17**)

A nitrogen atmosphere was established in a three-neck flask containing the appropriate imidazolyl-ethanone (1 mmol) and [(*R*,*R*)-TsDPEN]Ru-(*p*-cymene)Cl (0.001 mmol) before the addition of dichloromethane (7 mL) and Et_3_N (5 mmol). Formic acid (5 mmol) was added over a period of an hour. The mixture was stirred at room temperature for 26 h. NaHCO_3_ saturated solution was added cautiously and the organic layer with water and brine and dried over anhydrous Na_2_SO_4_. The solvent was removed under vacuum, affording the desired product. For each compound, imidazolyl-ethanone, melting point (°C), yield (%), IR, ^1^H NMR, and elemental analysis are reported.

### General Procedure D (GP-D) to Obtain Esters and Ethers (**3a**–**5a**, **3b**–**6b**, **7**, and **8b**–**10b**)

To a stirred suspension of the proper alcohol (0.46 mmol), in dry acetonitrile (6.0 mL), 0.46 mmol of sodium hydride was added at room temperature (or −5 °C for compounds **8b**–**10b**). The reaction mixture was stirred at room temperature for 2 h, then the appropriate benzoyl chloride (0.64 mmol) (or the proper benzyl halide (0.46 mmol) at 0 °C) was added, and the reaction was stirred for a further 24 h at reflux (or at room temperature for compounds **8b**–**10b**). The solvent was removed under reduced pressure, and the residue was dissolved in dichloromethane and washed with aqueous saturated potassium carbonate (or water for compounds **8b**–**10b**). The organic layer was dried over anhydrous Na_2_SO_4_ and, after filtration, was evaporated under reduced pressure. The crude residue was purified by column chromatography on silica gel using DCM/MeOH/*n*-hexane (9:1:0.5) or DCM/MeOH (9.5:0.5). For each compound, alcohol, benzoyl chloride, yield (%), ee (%), IR, ^1^H NMR, MS (ESI), and elemental analysis are reported.

#### (*R*)-2-(1*H*-Imidazol-1-yl)-1-(4-methoxyphenyl)ethyl 3-(Trifluoromethyl)benzoate (**3a**)

Compound **3a** was prepared from **11a** and 3-(trifluoromethyl)benzoyl chloride by means of GP-D. 26% as a brown wax; e.e. 94.3%; IR ν C=O 1723 cm^–1^; ^1^H NMR (CD_3_CN, δ) 3.80 (s, 3H, OCH_3_), 4,43 (dd, *J* = 4.2 Hz, *J* = 14.6 Hz, 1H, CH_a_), 4.55 (dd, *J* = 8.2 Hz, *J* = 14.6 Hz, 1H, CH_b_); 6.21 (dd, *J* = 4.2 Hz, *J* = 8.2 Hz, 1H, CH); 6.88 (s, 1H, imidazole); 6.95–6.97 (m, 2H, Ar); 7.08 (s, 1H, imidazole); 7.39–7.41 (m, 2H, Ar); 7.47 (s, 1H, imidazole); 7.73 (t, *J* = 7.8 Hz, 1H, Ar); 7.96 (d, *J* = 7.8 Hz, 1H, Ar); 8.29–8.32 (m, 2H, Ar). ^13^C NMR (CD_3_CN, δ): 164.42, 160.62, 138.40, 133.68, 131.32, 131.20, 131.04 (q, *J* = 33 Hz), 130.51 (q, *J* = 4 Hz), 129.55, 129.14, 128.62, 126.63 (q, *J* = 4 Hz), 124.49 (q, *J* = 270 Hz), 120.43, 114.61, 76.28, 55.56, 51.39. MS *m*/*z* (ESI) calcd for [C_20_H_17_F_3_N_2_O_3_]^+^ 390.1, found 391.4. Anal. Calcd for C_20_H_17_F_3_N_2_O_3_: C, 61.54; H, 4.39; N, 7.19%. Found: C, 61.79; H, 4.12; N, 6.99%.

#### (*S*)-2-(1*H*-Imidazol-1-yl)-1-(4-methoxyphenyl)ethyl 3-(Trifluoromethyl)benzoate (**3b**)

Compound **3b** was prepared from **11b** and 3-(trifluoromethyl)benzoyl chloride by means of GP-D. 11% as a brown wax; e.e. 95.1%; IR ν C=O 1726 cm^–1^;^1^H NMR (CD_3_CN, δ) 3.77 (s, 1H, OCH_3_), 4.43 (dd, *J* = 3.8 Hz, *J* = 14.6 Hz, 1H, CH_a_), 4.55 (dd, *J* = 7.4 Hz, *J* = 14.6 Hz, 1H, CH_b_), 6.19 (dd, *J* = 3.8 Hz, *J* = 7.4 Hz, 1H, CH), 6.91–6.95 (m, 3H, Ar and imidazole), 7.09 (s, 1H, imidazole), 7.36–7.38 (m, 2H, Ar), 7.56 (s, 1H, imidazole), 7.70 (t, *J* = 7.8 Hz, 1H, Ar), 7.93 (d, *J* = 7.8 Hz, 1H, Ar), 8.23–8.29 (m, 3H, Ar). ^13^C NMR (CD_3_CN, δ): 164.41, 160.61, 138.41, 133.68, 131.32, 131.20, 131.04 (q, *J* = 33 Hz), 130.51 (q, *J* = 4 Hz), 129.55, 129.15, 128.62, 126.63 (q, *J* = 4 Hz), 124.49 (q, *J* = 270 Hz), 120.43, 114.61, 76.28, 55.54, 51.39. MS *m*/*z* (ESI) calcd for [C_20_H_17_F_3_N_2_O_3_]^+^ 390.1, found 391.1. Anal. Calcd for C_20_H_17_F_3_N_2_O_3_: C, 61.54; H, 4.39; N, 7.19%. Found: C, 61.27; H, 4.58; N, 7.01%.

#### (*R*)-1-(4-Fluorophenyl)-2-(1*H*-imidazol-1-yl)ethyl 3,5-Dichlorobenzoate (**4a**)

Compound **4a** was prepared from **12a** and 3,5-dichlorobenzoyl chloride by means of GP-D. 21 % as brown wax; e.e. 82.3%; IR ν C=O 1729 cm^–1^;^1^H NMR (CD_3_CN, δ) 4.44 (dd, *J* = 3.3 Hz, *J* = 14.3 Hz, 1H, CH_a_), 4.53 (dd, *J* = 8.9 Hz, *J* = 14.4 Hz, 1H, CH_b_), 6.23 (dd, *J* = 3.3 Hz, *J* = 8.6 Hz, 1H, CH), 6.89 (s, 1H, imidazole), 7.06 (s, 1H, imidazole), 7.16 (m, 2H, Ar), 7.47–7.50 (m, 3H, Ar and imidazole), 7.76 (t, *J* = 7.8 Hz, 1H, Ar), 7.98 (d, *J* = 7.9 Hz, 1H, Ar), 8.30–8.34 (m, 2H, Ar). ^13^C NMR (CD_3_OD, δ): 163.06 (d, *J* = 245 Hz), 162.70, 137.90, 135.43, 132.95, 132.62, 132.60 (d, *J* = 3 Hz), 132.00, 129.12, 128.50 (d, *J* = 8 Hz), 120.03, 115.34 (d, *J* = 22 Hz), 75.48, 50.79. MS *m*/*z* (ESI) calcd for [C_18_H_13_Cl_2_FN_2_O_2_]^+^ 378.0, found 379.3. Anal. Calcd for C_18_H_13_Cl_2_FN_2_O_2_: C, 57.01; H, 3.46; N, 7.39%. Found: C, 56.87; H, 3.58; N, 7.05%.

#### (*S*)-1-(4-Fluorophenyl)-2-(1*H*-imidazol-1-yl)ethyl 3,5-Dichlorobenzoate (**4b**)

Compound **4b** was prepared from **12b** and 3,5-dichlorobenzoyl chloride by means of GP-D. 10% as brown wax; e.e. 81.4%; IR ν C=O 1729 cm^–1^;^1^H NMR (DMSO-*d*_6_, δ) 4.43 (dd, *J* = 3.6 Hz, *J* = 14.4 Hz, 1H, CH_a_), 4.54 (dd, *J* = 8.8 Hz, *J* = 14.3 Hz, 1H, CH_b_), 6.22–6.24 (m 1H, CH), 6.89 (s, 1H, imidazole), 7.07 (s, 1H, imidazole), 7.14–7.18/ (m, 2H, Ar), 7.47–7.50 (m, 3H, Ar), 7.75 (s, 1H, imidazole), 7.99 (s, 2H, Ar). ^13^C NMR (Acetone-d_6_, δ): 162.81 (d, *J* = 244 Hz), 162.54, 137.89, 135.25, 133.28 (d, *J* = 3 Hz), 133.27, 132.99, 128.90 (d, *J* = 8 Hz), 128.73, 127.96, 119.71, 115.47 (d, *J* = 22 Hz), 75.84, 50.71. MS *m*/*z* (ESI) calcd for [C_18_H_13_Cl_2_FN_2_O_2_]^+^ 378.0, found 379.3. Anal. Calcd for C_18_H_13_Cl_2_FN_2_O_2_: C, 57.01; H, 3.46; N, 7.39%. Found: C, 56.78; H, 3.67; N, 7.12%.

#### (*R*)-1-(2,4-Dichlorophenyl)-2-(1*H*-imidazol-1-yl)ethyl 3-(Trifluoromethyl)benzoate (**5a**)

Compound **5a** was prepared from **13a** and 3-(trifluoromethyl)benzoyl chloride by means of GP-D. 25% as pale brown wax; e.e. 82.2%; IR ν C=O 1729 cm^–1^;^1^H NMR (DMSO-*d*_6_, δ) 4.53 (d, *J* = 5.2 Hz, 2H, CH_a_H_b_), 6.49 (t, *J* = 5.6 Hz, 1H, CH), 6.88 (s, 1H, imidazole), 7.02 (s, 1H, imidazole), 7.31 (s, 2H, Ar), 7.42 (s, 1H, Ar), 7.57–7.59 (m, 1H, Ar), 7.72–7.76 (m, 1H, Ar), 7.97–7.99 (m, 1H, Ar), 8.31–8.33 (m, 2H, Ar). ^13^C NMR (CD_3_CN, δ): 164.20, 138.46, 135.28, 134.08, 133.84, 133.35, 131.11 (q, *J* = 32 Hz), 130.80 (q, *J* = 4 Hz), 130.79, 130.55, 129.85, 129.40, 129.37, 128.38, 126.80 (q, *J* = 4 Hz), 124.42 (q, *J* = 270 Hz), 120.59, 73.02, 49.71. MS *m*/*z* (ESI) calcd for [C_19_H_13_Cl_2_F_3_N_2_O_2_]^+^ 428.0, found 429.5. Anal. Calcd for C_19_H_13_Cl_2_F_3_N_2_O_2_: C, 53.17; H, 3.05; N, 6.53%. Found: C, 52.89; H, 3.38; N, 6.67%.

#### (*S*)-1-(2,4-Dichlorophenyl)-2-(1*H*-imidazol-1-yl)ethyl 3-(Trifluoromethyl)benzoate (**5b**)

Compound **5b** was prepared from **13b** and 3-(trifluoromethyl)benzoyl chloride by means of GP-D. 7% as dark green wax; e.e. 82.2%; IR ν C=O 1728 cm^–1^;^1^H NMR (DMSO-*d*_6_, δ) 4.55 (d, *J* = 5.2 Hz, 2H, CH_a_H_b_), 6.52 (t, *J* = 5.6 Hz, 1H, CH), 6.90 (s, 1H, imidazole), 7.04 (s, 1H, imidazole), 7.34 (s, 2H, Ar), 7.44 (s, 1H, Ar), 7.59 (s, 1H, imidazole), 7.77–7.79 (m, 1H, Ar), 8.00–8.02 (m, 1H, Ar), 8.34–8.36 (m, 2H, Ar. ^13^C NMR (CD_3_CN, δ): 164.22, 138.40, 135.33, 134.03, 133.87, 133.38, 131.04 (q, *J* = 32 Hz), 130.80 (q, *J* = 4 Hz), 130.79, 130.56, 129.89, 129.42, 128.89, 128.41, 126.82 (q, *J* = 4 Hz), 124.45 (q, *J* = 270 Hz), 120.78, 72.94, 49.84. MS *m*/*z* (ESI) calcd for [C_19_H_13_Cl_2_F_3_N_2_O_2_]^+^ 428.0, found 429.1. Anal. Calcd for C_19_H_13_Cl_2_F_3_N_2_O_2_: C, 53.17; H, 3.05; N, 6.53%. Found: C, 53.05; H, 3.28; N, 6.76%.

#### (*S*)-1-(2,4-Dichlorophenyl)-2-(1*H*-imidazol-1-yl)ethyl 2,4-Dichlorobenzoate (**6b**)

Compound **6b** was prepared from **13b** and 2,4-dichlorobenzoyl chloride by means of GP-D. 20% as yellow wax; e.e. 94.6%; IR ν C=O 1732 cm^–1^; ^1^H NMR (CD_3_CN, δ) 4.49 (d, *J* = 5.2 Hz, 2H, CH_a_H_b_,), 6.47 (t, *J* = 5.2 Hz, 1H, CH), 6.87 (s, 1H, imidazole), 6.97 (s, 1H, imidazole), 7.27–7.32 (m, 2H, Ar), 7.38 (s, 1H, imidazole), 7.47 (dd, *J* = 8.5, *J* = 1.9, 1H, Ar), 7.56 (d, *J* = 1.6 Hz, 1H, Ar), 7.61 (d, *J* = 1.9 Hz, 1H, Ar), 7.91 (d, *J* = 8.5 Hz, 1H, Ar). ^13^C NMR (CD_3_CN, δ): 162.95, 138.67, 134.74, 134.63, 133.37, 133.00, 132.86, 131.00, 129.29, 128.94, 128.73, 127.82, 127.57, 127.36, 72.69, 49.13. MS *m*/*z* (ESI) calcd for [C_18_H_12_Cl_4_N_2_O_2_]^+^ 428.0, found 429.4. Anal. Calcd for C_18_H_12_Cl_4_N_2_O_2_: C, 50.27; H, 2.81; N, 6.51%. Found: C, 50.25; H, 2.81; N, 6.50%.

#### (*S*)-1-(4-Fluorophenyl)-2-(1*H*-1,2,4-triazol-1-yl)ethyl 3-(Trifluoromethyl)benzoate (**7**)

Compound **7** was prepared from **17** and 3-(trifluoromethyl)benzoyl chloride by means of GP-D. 21% as colorless wax; IR ν C=O 1726 cm^–1^; ^1^H NMR (CD_3_CN, δ) 4.67 (dd, *J* = 3.5 Hz, *J* = 14.2 Hz, 1H, CH_a_), 4.82 (dd, *J* = 8.3 Hz, *J* = 14.2 Hz, 1H, CH_b_), 6.9 (dd, *J* = 3.5 Hz, *J* = 8.3 Hz, 1H, CH), 7.16 (t, *J* = 8.7 Hz, 2H, Ar), 7,50–7.54 (m, 2H, Ar), 7.69–7.74 (m, 1H, Ar), 7.88 (s, 1H, Ar), 7.93–7.97 (m, 1H, Ar), 8.19 (s, 1H, triazole), 8.22–8.29 (m, 1H, Ar), 8.33 (s, 1H, triazole). ^13^C NMR (CD_3_CN, δ) 163.82, 162.86 (d, *J* = 243 Hz), 151.78, 144.74, 133.21, 132.94 (d, *J* = 3 Hz), 130.64, 130.52 (q, *J* = 33 Hz), 130.07 (q, *J* = 4 Hz), 129.91, 128.90 (d, *J* = 9 Hz), 126.19 (q, *J* = 4 Hz), 123.96 (q, *J* = 269 Hz), 115.62 (d, *J* = 22 Hz), 74.31, 53.28. MS *m*/*z* (ESI) calcd for [C_18_H_13_F_4_N_3_O_2_]^+^ 379.1, found 379.8. Anal. Calcd for C_18_H_13_F_4_N_3_O_2_: C, 57.00; H, 3.45; N, 11.08%. Found: C, 57.31; H, 3.28; N, 11.37%.

#### (*S*)-1-(2-((3,5-Dichlorobenzyl)oxy)-2-(4-fluorophenyl)ethyl)-1*H*-imidazole (**8b**)

Compound **8b** was prepared from **12b** and 3,5-dichlorobenzyl chloride by means of GP-E. 21% as yellow wax; ee 95.1%; IR ν C–O–C 1077, 1225 cm^–1^; ^1^H NMR (CD_3_CN, δ) 4.15 (dd, *J* = 4.0, *J* = 14.4, 1H, N–CH_a_), 4.19–4.25 (m, 2H, N–CH_b_ + O–CH_a_), 4.39 (d, 1H, O–CH_b_). 4.66 (dd, *J* = 4.0, *J* = 7.6, 1H, CH), 6.90 (s, 1H, imidazole), 6.99 (s, 1H, imidazole), 7.10–7.15 (m, 4H, Ar), 7.33–7.39 (m, 4H, Ar and imidazole). ^13^C NMR (CD_3_CN, δ): 163.23 (d, *J* = 243 Hz),142.88, 138.42, 135.09, 135.05(d, *J* = 3 Hz), 129.49 (d, *J* = 8 Hz),128.93, 127.79, 126.27, 120.46, (d, *J* = 21 Hz), 80.59, 69.40, 52.78. MS *m*/*z* (ESI) calcd for [C_18_H_15_Cl_2_FN_2_O]^+^ 364.0, found 365.2. Anal. Calcd for C_18_H_15_Cl_2_FN_2_O: C, 59.20; H, 4.14; N, 7.67%. Found: C, 59.22; H, 4.14; N, 7.66%.

#### (*S*)-1-(2-(2,4-Dichlorophenyl)-2-((3-(trifluoromethyl)benzyl)oxy)ethyl)-1*H*-imidazole (**9b**)

Compound **9b** was prepared from **13b** and 3-(trifluoromethyl)benzyl bromide by means of GP-E. 6% as yellow wax; e.e. 81.4%; IR ν C–O–C 1044, 1124 cm^–1^; ^1^H NMR (CD_3_CN, δ) 4.19 (dd, *J* = 7.9 Hz, *J* = 14.6 Hz, 1H, N–CH_a_), 4.27 (dd, *J* = 3.6 Hz, *J* = 14.6 Hz, 1H, N–H_b_), 4.38 (d, *J* = 12.3 Hz, 1H, O–CH_a_), 4.52 (d, *J* = 12.3 Hz, 1H, O–CH_b_), 5.06 (dd, *J* = 3.6 Hz, *J* = 7.12 Hz, 1H, CH), 6.88 (s, 1H, imidazole), 6.96 (s, 1H, imidazole), 7.34–7.39 (m, 3H, Ar), 7.44–7.53 (m, 4H, Ar and imidazole), 7.59 (d, *J* = 7.4, 1H, Ar). ^13^C NMR (CD_3_OD, δ): 140.11, 139.14 (q, *J* = 2 Hz), 136.04, 135.66, 134.88, 132.43 (q, *J* = 2 Hz), 131.77 (q, *J* = 32 Hz), 130.49, 130.34, 130.33, 129.04, 128.65, 125.62 (q, *J* = 4 Hz), 125.56 (q, *J* = 271 Hz), 125.38 (q, *J* = 4 Hz), 78.16, 71.69, 52.14. MS *m*/*z* (ESI) calcd for [C_18_H_15_Cl_2_FN_2_O]^+^ 414.0, found 415.8. Anal. Calcd for C_19_H_15_Cl_2_F_3_N_2_O: C, 54.96; H, 3.64; N, 6.75%. Found: C, 54.94; H, 3.64; N, 6.76%.

#### (*S*)-1-(2-((3,5-Dichlorobenzyl)oxy)-2-(2,4-dichlorophenyl)ethyl)-1*H*-imidazole (**10b**)

Compound **10b** was prepared from **13b** and 3,5-dichlorobenzyl chloride by means of GP-E. 44% as colorless wax; e.e. 92.6%; IR ν C–O–C 1094, 1231 cm^–1^; ^1^H NMR (CD_3_CN, δ) 4.32 (dd, *J* = 7,3, *J* = 14.6, 1H, N–CH_a_), 4.39 (dd, *J* = 3,7, *J* = 14.6, 1H, N–CH_b_), 4.41 (dd, *J* = 12.7, 1H, O–CH_a_), 4.54 (dd, *J* = 12.8, 1H, O–CH_b_), 5.14 (dd, *J* = 3.6, *J* = 7.2, 1H, CH), 6.90 (s, 1H, imidazole), 7.05 (s, 1H, Ar), 7.36 (s, 1H, Ar), 7.41–7.44 (m, 1H, Ar), 7.48–7.50 (m, 2H, Ar + imidazole), 7.54 (d, 1H, *J* = 2.0 Hz, Ar). ^13^C NMR (CD_3_CN, δ): 142.32, 138.42, 135.14, 135.13, 134.82, 134.12, 129.81, 129.72, 129.06, 128.33, 127.99, 126.49, 120.49, 77.62, 66.99, 51.11. MS *m*/*z* (ESI) calcd for [C_18_H_14_Cl_4_N_2_O]^+^ 415.9, found 416.4. Anal. Calcd for C_18_H_14_Cl_4_N_2_O: C, 51.96; H, 3.39; N, 6.73%. Found: C, 51.94; H, 3.39; N, 6.72%.

#### (*R*)-2-(1*H*-Imidazol-1-yl)-1-(4-methoxyphenyl)ethan-1-ol (**11a**)

Compound **11a** was prepared from **14** by means of GP-B. 193–195 °C; 31% as white solid; IR ν OH 3123 cm^–1^; ^1^H NMR (DMSO-*d*_6_, δ) 3.74 (s, 3H, OCH_3_), 4.00 (dd, *J* = 8.0 Hz, *J* = 14 Hz, 1H, CH_a_H_b_), 4.09 (dd, *J* = 4.4 Hz, *J* = 14 Hz, 1H, CH_a_H_b_), 4.75 (m, 1H, CH), 5.60 (d, *J* = 4.4 Hz, 1H, OH), 6.82 (s, 1H, imidazole), 6.88 (d, *J* = 7.8 Hz, 2H, Ar), 7.10 (s, 1H, imidazole), 7.25 (d, *J* = 8.8 Hz, 2H, Ar), 7.48 (s, 1H, imidazole). MS *m*/*z* (ESI) calcd for [C_18_H_14_Cl_4_N_2_O]^+^ 218.1, found 219.1. Anal. Calcd for C_12_H_14_N_2_O_2_: C, 66.04%; H, 4.67; N, 12.84%. Found: C, 65.88; H, 4.82; N, 12.57%.

#### (*S*)-2-(1*H*-Imidazol-1-yl)-1-(4-methoxyphenyl)ethan-1-ol (**11b**)

Compound **11b** was prepared from **14** by means of GP-C. 188–190 °C; 45% as white solid; IR ν OH 3123 cm^–1^; ^1^H NMR (DMSO-*d*_6_, δ) 3.74 (s, 3H, OCH_3_), 4.01 (dd, *J* = 7.8 Hz, *J* = 13.8 Hz, 1H, CH_a_H_b_), 4.09 (dd, *J* = 4.2 Hz, *J* = 13.7 Hz, 1H, CH_a_H_b_), 4.75–4.77 (m, 1H, CH), 5.58 (bs, 1H, OH), 6.82–6.90 (m, 3H, Ar and imidazole), 7.10 (s, 1H, imidazole), 7.24–7.26 (m, 2H, Ar), 7.47 (s, 1H, imidazole). MS *m*/*z* (ESI) calcd for [C_18_H_14_Cl_4_N_2_O]^+^ 218.1, found 218.3. Anal. Calcd for C_12_H_14_N_2_O_2_: C, 66.04; H, 4.67; N, 12.84%. Found: C, 65.92; H, 4.79; N, 12.75%.

#### (*R*)-1-(4-Fluorophenyl)-2-(1*H*-imidazol-1-yl)ethan-1-ol (**12a**)

Compound **12a** was prepared from **15** according to the literature^50,51^ by means of GP-B. 170–172 °C; 31% as pink solid; IR ν OH 3115 cm^–1^; ^1^H NMR (CD_3_OD, δ) 4.18 (dd, *J* = 6.8 Hz, *J* = 14 Hz, 1H, CH_a_H_b_), 4.23 (dd, *J* = 4.4 Hz, *J* = 14 Hz, 1H, CH_a_H_b_), 4.94 (dd, *J* = 4.4 Hz, *J* = 6.8 Hz, 1H, CH), 6.92 (s, 1H, imidazole), 7.09–7.04 (m, 3H, Ar and imidazole), 7.36–7.33 (m, 2H, Ar), 7.51 (s, 1H, imidazole). MS *m*/*z* (ESI) calcd for [C_11_H_11_FN_2_O]^+^ 206.1, found 207.3. Anal. Calcd for C_11_H_11_FN_2_O: C, 64.07; H, 5.38; N, 13.58%. Found: C, 63.98; H, 5.47; N, 13.22%.

#### (*S*)-1-(4-Fluorophenyl)-2-(1*H*-imidazol-1-yl)ethan-1-ol (**12b**)

Compound **12b** was prepared from **15** according to the literature^50,51^ by means of GP-C. 173–175 °C; 58% as pink solid; IR ν OH 3116 cm^–1^; ^1^H NMR (CD_3_CN, δ) 3.90 (bs, 1H, OH), 4.10 (dd, *J* = 7.3 Hz, *J* = 14.1 Hz, 1H, CH_a_H_b_), 4.15 (dd, *J* = 4.5, *J* = 14.1 Hz, 1H, CH_a_H_b_), 4.93 (m, 1H, CH), 6.89 (s, 1H, imidazole), 7.00 (s, 1H, imidazole), 7.08–7.13 (m, 2H, Ar), 7.34–7.37 (m, 3H, Ar and imidazole). MS *m*/*z* (ESI) calcd for [C_11_H_11_FN_2_O]^+^ 206.1, found 207.2. Anal. Calcd for C_11_H_11_FN_2_O: C, 64.07; H, 5.38; N, 13.58%. Found: C, 64.22; H, 5.12; N, 13.15%.

#### (*R*)-1-(2,4-Dichlorophenyl)-2-(1*H*-imidazol-1-yl)ethan-1-ol (**13a**)

Compound **13a** was prepared from **16** according to the literature^50,51^ by means of GP-B. 90–93 °C; 88% as pale yellow solid; IR ν OH 3111 cm^–1^; ^1^H NMR (DMSO-*d*_6_, δ) 4.05 (dd, *J* = 7.1 Hz, *J* = 14.1 Hz, 1H, CH_a_H_b_), 4.18 (dd, *J* = 3.4, *J* = 14.2 Hz, 2H, CH_a_H_b_), 5.08 (m, 1H, CH), 6.03 (s,1H, OH), 6.84 (s, 1H imidazole), 7.05 (s, 1H, imidazole), 7.42- 7.48 (m, 3H, Ar and imidazole), 7.60 (s, 1H, *J* = 1.6 Hz, Ar). MS *m*/*z* (ESI) calcd for [C_11_H_10_Cl_2_N_2_O]^+^ 256.0, found 257.1. Anal. Calcd for C_11_H_10_Cl_2_N_2_O: C, 51.38; H, 3.92; N, 10.90%. Found: C, 51.53; H, 3.85; N, 10.72%.

#### (*S*)-1-(2,4-Dichlorophenyl)-2-(1*H*-imidazol-1-yl)ethan-1-ol (**13b**)

Compound **13b** was prepared from **16** according to the literature^50,51^ by means of GP-C. 97–100 °C; 33% as yellow solid; IR ν OH 3113 cm^–1^; ^1^H NMR (DMSO-*d*_6_, δ) 4.05 (dd, *J* = 7.1 Hz, *J* = 14.1 Hz, 1H, CH_a_H_b_), 4.18 (dd, *J* = 3.4 Hz, *J* = 14.2 Hz, 1H, CH_a_H_b_), 5.07–5.09 (m, 1H, CH), 6.04 (bs, 1H, OH), 6.86 (s, 1H, imidazole), 7.05 (s, 1H, imidazole), 7.42–7.48 (m, 3H, Ar and imidazole), 7.60 (d, *J* = 1.8 Hz, 1H, Ar). MS *m*/*z* (ESI) calcd for [C_11_H_10_Cl_2_N_2_O]^+^ 256.0, found 257.3. Anal. Calcd for C_11_H_10_Cl_2_N_2_O: C, 51.38; H, 3.92; N, 10.90%. Found: C, 51.67; H, 3.82; N, 10.55%.

#### 2-(1*H*-Imidazol-1-yl)-1-(4-methoxyphenyl)ethan-1-one (**14**)

Compound **14** was prepared from 2-bromo-1-(4-methoxyphenyl)ethan-1-one^57^ by means of GP-A. 126–128 °C; 48% as pale yellow solid; IR ν C=O 1680 cm^–1^; ^1^H NMR (DMSO-*d*_6_, δ) 3.88 (s, 3H, OCH_3_), 5.67 (s, 2H, CH_2_), 6,91 (s, 1H, imidazole), 7.11–7.13 (m, 3H, Ar and imidazole), 7.58 (s, 1H, imidazole), 8.01–8.03 (m, 2H, Ar). MS *m*/*z* (ESI) calcd for [C_12_H_12_N_2_O_2_]^+^ 216.1, found 217.2. Anal. Calcd for C_12_H_12_N_2_O_2_: C, 66.65; H, 5.59; N, 12.96%. Found: C, 66.71; H, 5.55; N, 12.88%.

#### 1-(4-Fluorophenyl)-2-(1*H*-imidazol-1-yl)ethan-1-one (**15**)

Compound **15** was prepared from 2-bromo-1-(4-fluorophenyl)ethan-1-one according to the literature^43^ by means of GP-A. 145–147 °C; 48% as white solid; IR ν C=O 1698 cm^–1^; ^1^H NMR (CD_3_CN, δ) 5.55 (s, 2H, CH_2_), 7.03 (s, 1H, imidazole), 7.12 (s, 1H, imidazole), 7.31–7.36 (m, 2H, Ar), 7.49 (s, 1H, imidazole), 8.07–8.13 (m, 2H, Ar). MS *m*/*z* (ESI) calcd for [C_11_H_9_FN_2_O]^+^ 204.1, found 205.2. Anal. Calcd for C_11_H_9_FN_2_O: C, 64.70; H, 4.44; N, 13.72%. Found: C, 64.58; H, 4.77; N, 13.48%.

#### 1-(2,4-Dichlorophenyl)-2-(1*H*-imidazol-1-yl)ethan-1-one (**16**)

Compound **16** was prepared from 2-bromo-1-(2,4-dichlorophenyl)ethan-1-one^58^ by means of GP-A. 60–61 °C; 57% as brown solid; IR ν C=O 1713 cm^–1^; ^1^H NMR (CD_3_CN, δ) 5.41 (s, 2H, CH_2_), 6.99 (s, 1H, imidazole), 7.05 (s, 1H, imidazole), 7,51–7.54 (m, 2H, Ar and imidazole), 7.66 (m, 1H, Ar), 7.74 (d, *J* = 8.4 Hz, 1H, Ar). MS *m*/*z* (ESI) calcd for [C_11_H_8_Cl_2_N_2_O]^+^ 254.0, found 255.3. Anal. Calcd for C_11_H_8_Cl_2_N_2_O: C, 51.79; H, 3.16; N, 10.98%. Found: C, 51.87; H, 3.22; N, 11.06%.

#### (*S*)-1-(4-Fluorophenyl)-2-(1*H*-1,2,4-triazol-1-yl)ethan-1-ol (**17**)

Compound **17** was prepared from **18** by means of GP-C. 111–112 °C; 40% as pink solid; IR ν OH 3207 cm^–1^; ^1^H NMR (DMSO-*d*_6_, δ) 4.29–4.32 (m, 2H, CH_2_), 4.95 (m, 1H, CH), 5.81 (d, *J* = 5 Hz, 1H, OH), 7.14–7.18 (m, 2H, Ar), 7.36–7.39 (m, 2H, Ar), 7.94 (s, 1H, triazole), 8.36 (s, 1H, triazole). MS *m*/*z* (ESI) calcd for [C_10_H_10_FN_3_O]^+^ 207.1, found 208.0. Anal. Calcd for C_10_H_10_FN_3_O: C, 57.97; H, 4.86; N, 20.28%. Found: C, 58.23; H, 4.47; N, 20.06%.

#### 1-(4-Fluorophenyl)-2-(1*H*-1,2,4-triazol-1-yl)ethan-1-one (**18**)

Compound **18** was prepared from 2-bromo-1-(4-fluorophenyl)ethan-1-one by means of GP-A. 125–127 °C; 57% as brown solid; IR ν C=O 1687 cm^–1^; ^1^H NMR (DMSO-*d*_6_, δ) 5.99 (s, 2H, CH_2_), 7.44 (t, *J* = 8.0 Hz, 2H, Ar), 8.00 (s, 1H, triazole), 8.15 (dd, *J* = 4.0 Hz, *J* = 12.0 Hz, 1H, Ar), 8.51 (s, 1H, triazole). MS *m*/*z* (ESI) calcd for [C_10_H_8_FN_3_O]^+^ 205.1, found 206.3. Anal. Calcd for C_10_H_8_FN_3_O: C, 58.53; H, 3.93; N, 20.48%. Found: C, 58.62; H, 3.99; N, 20.68%.

### Compound Evaluation in the *Naegleria* Cell-Based Assay

The compounds were screened against *N. fowleri* European KUL strain axenically cultured in Nelson’s medium supplemented with 10% fetal bovine serum at 37 °C.^59^ The screening assay in 96- and 384-well formats (*Z*’-value of 0.95 ± 0.1) was performed as described elsewhere.^9^ All the experiments were performed using trophozoites harvested during the logarithmic phase of growth.^60^ The primary screen was performed in duplicate at 10 μM in a 384-well format (2,500 amoebae/well). The dose–response curves were generated in triplicate for the follow up compounds by serial dilution of compounds from 50 to 0.39 μM in a 96-well plate with 10,000 amoebae/well. Assay plates were incubated for 48 h at 37 °C, and cell viability was determined by a CellTiter-Glo Luminescent Cell Viability Assay.^9,58^ The experiments were conducted in a biosafety cabinet following BSL-2 procedures as specified in the UCSD Biosafety Practices Guidelines.

### Binding Titrations of NfCYP51

Spectral binding titrations of NfCYP51 with the test compounds were performed at 25 °C with 0.5 μM NfCYP51. The concentration of NfCYP51 was determined for the ferrous (reduced) carbon monoxide (CO)-bound species at 450 nm (ε = 91,000 M^–1^ cm^–1^) that represents an active protein fraction with the intact heme Fe thiolate bond. All the inhibitors were dissolved in 100% DMSO as 100 and 200 μM stock solutions. Miconazole and fluconazole were used as references. For each titration, 4 mL of NfCYP51 (in 50 mM potassium phosphate, pH 8.0, and 10% glycerol) was split equally between the reference and sample plastic UV-cuvettes (Cat.#67.758; Sarstedt, Germany). Inhibitor aliquots of 0.5 μL were successively added to the sample cuvette in 25 nM (first eight aliquots) and 50 nM (last six aliquots) increments in a concentration range of 0.025–0.5 μM; DMSO alone was added to the reference cuvette, with the total added volume being less than 1% of the sample volume. Spectra were recorded from 350 to 500 nm. A binding isotherm was generated by plotting a difference between the absorbance maximum at 430 nm and the absorbance minimum at 410 nm as a function of inhibitor concentration. The data were analyzed in GraphPad Prism 9 with the rearrangement of the Morrison binding equation^61^ to determine the dissociation constants *K*_*D*_: Δ*A* = (Δ*A*_max_/2[E])((*K*_D_ + [L] + [E]) – ((*K*_D_ + [E] + [L])^2^ – 4[E][L])^0.5^), where Δ*A* is the difference between the absorbance maximum and minimum, Δ*A*_max_ is the extrapolated maximum absorption difference, [L] is the ligand (inhibitor) concentration, and [E] is the enzyme concentration.

### X-ray Crystallography

Prior to crystallization, NfCYP51 at 0.5 mM concentration in storage buffer (50 mM potassium phosphate, pH 8.0) was incubated with 1.2 molar excess of the respective ligand for 30 min on ice. Crystals were then set up in 96-well plates using a hanging drop crystallization protocol and Mosquito liquid pipetting robot (STP LabTech, Boston, MA). We have used crystallization conditions similar to our report for NfCYP51 previously: 30 mM CaCl_2_; 33% v/v PEG MME 550; 100 mM bis-Tris propane, pH 7.0; concentration of Jefframine M-600 varied from 3 to 4.6%.^15^ The plates were configured by using the Dragonfly liquid pipetting robot equipped with the Designer software (STP LabTech, Boston, MA). All the crystals were obtained from 23 °C. Diffraction data were collected remotely at beamline 8.3.1, Advanced Light Source, Lawrence Berkeley National Laboratory. Data indexing, integration, and scaling were conducted using XDS.^62^ The structures were determined by molecular replacement using the NfCYP51–posaconazole complex (PDB 5TL8) as a molecular replacement model. The final models were built and refined using the BUCCANEER and REFMAC5 modules of the CCP4 software suite^63^ and COOT software.^64^ Data collection and refinement statistics are listed in Table 2.

### *In Vivo* BBB Permeability

All mice were maintained on a 12 h light/dark cycle in a temperature-controlled environment with access to food and water *ad libitum*. Mice were randomly divided into the following three groups (*N* = 2 each): (1) miconazole, mice were treated intraperitoneally (i.p.) with 40 mg/kg miconazole; (2) **8b**, mice were treated i.p. with 40 mg/kg **8b**; (3) **9b**, mice were treated i.p. with 40 mg/kg **9b**. Miconazole, **8b**, and **9b** were dissolved in 150 μL of DMSO. Mice were sacrificed 1 h following the drug administration, and blood and brain samples were collected. Blood (100 μL) was quickly added with 5-fold H_2_O/CH_3_CN 8:2, centrifugated to 2500 rpm for 15 min, and plasma was collected. Brain was homogenized in 0.5 mL of distilled water and added with 2 mL of CH_3_CN. Thus, homogenates were centrifugated to 2500 rpm for 15 min and the supernatants were collected. Plasma and brain supernatants were stored at −20 °C until analysis.

#### Ethics Statements

All experiments involving animals were carried out according to Sapienza University’s Ethics Committee, approval code 890/2021-PR, approved on 17 November 2021. Animal care followed the IASP and European Community (EC L358/1, 18/12/86) guidelines on using and protecting animals in experimental research. Eight week-old female C57BL/6 mice were used for the experiments (Charles River, Lecco, Italy).

### HPLC-ESI-MS/MS Analysis

#### HPLC-ESI-MS/MS Instrumental Conditions

The targeted analysis was performed by a Waters system composed of a 1525 μ HPLC (Milford, MA, USA), coupled with a Quattro Micro Tandem MS/MS with an ESI source (Micromass, Manchester UK), using a Supelco Ascentis Express C18 (15 cm × 2.1 mm) 2.7 μm analytical column, A (deionized water/formic acid 0.02%) and B (acetonitrile/formic acid 0.02%) as mobile phase, with the following optimized elution binary gradient with linear interpolation: 0–1 min, 30% B; 1–16 min, 45% B; 16–17 min, 45% B; 17–18 min, 30% B; 18–38 min, 30% B to equilibrate the column, flow rate 0.20 mL min^–1^. Infusion experiments in positive ionization (ES+) were performed to optimize (a) the ESI source parameters and (b) the best fragmentation of each compound, **1**, **8b**, and **9b**, to choose the best transition for the multiple reaction monitoring (MRM) mass method. In detail, (a) capillary voltage 2700 V, cone voltage 22 V, source temperature 150 °C, desolvation temperature 350 °C, cone gas flow 30 L h^–1^, desolvation gas flow 400 L h^–1^ and (b) two transitions for MIC, 417 → 159 (*m*/*z*) and 417 → 161 (*m*/*z*), dwell cell value of 0.200 s; one transition for 368, 415 → 159 (*m*/*z*), dwell cell value of 0.200 s; one transition for 370, 365 → 159 (*m*/*z*), dwell cell value of 0.200 s were used. Data acquisition, data handling, and instrument control were performed by MassLynx Software 4.1 v (Data Handling System for Windows, Micromass, UK).

#### Calibration Standard Solution Preparation and Curve Calculation

Three stock solutions of **1**, **8b**, and **9b** were prepared by dissolving 1 mg mL^–1^ of each compound in methanol and stored at 4 °C. Each stock solution was appropriately diluted with the mobile phase (A/B, 70:30, v:v) and used to optimize the ESI source parameters and the MRM method. A working solution containing **1**, **8b**, and **9b** was then prepared by diluting 1:100 with the mobile phase (A/B, 70:30, v:v) equal aliquots of each compound stock solution, and it was used to optimize the chromatographic conditions.

Working solutions containing all compounds, **1**, **8b**, and **9b**, at the final concentrations of 2.5, 5.0, 10.0, 25.0, 35.0, and 50.0 ng mL^–1^ were prepared by appropriately diluting each compound stock solution with the mobile phase (A:B, 70:30 v:v) and analyzed in triplicate (25 μL injected) to construct the calibration curves. Calibration curves were calculated with equal weighted least-squares linear regression analysis of the MRM peak area against the standard nominal concentration. A very good linearity was found for all compounds in the analyzed range, with *R*^2^ values of 0.99 for all compounds, as shown in Table S2. The matrix effect (ME) for each compound, in brain (B) and in plasma (P), was evaluated by comparing the matrix-matching calibration curve (2.5, 10, 25, and 50 ng mL^–1^) with the corresponding calibration curve.^65^ ME values, reported in Table S2, were found in agreement with a generally weak matrix effect.^66^ Chromatographic (elution time *t*_R_) and mass spectral (MRM transition, dwell time) data are reported in Table S2 too. The calibration curves were used for the quantitation of each compound **1**, **8b**, and **9b** identified in the analyzed biological matrices.

#### Biological Sample Preparation

The biological samples were appropriately diluted with the mobile phase (A:B, 70:30, v:v), filtered at 0.22 μm, and analyzed in triplicate (25 μL injected). The amounts of compounds **1**, **8b**, and **9b**, quantitated in duplicate in brain and in blood samples are reported in Table 3 as mean values ± standard deviation of triplicate analysis.

## Supporting Information Available

Molecular formula strings and some data (CSV)
Hits with ≥50% inhibition (Table S1) and retention time (*t*_R_), dwell time, MRM transition, linear relation (*R*^2^), and matrix effect (ME) (Table S2), ^1^H NMR and ^13^C NMR spectra of compounds **3a**–**5a**, **3b**–**6b**, **7**, and **8b**–**10b**, HPLC traces of lead compounds **8b** and **9b**, PDB validation reports for new X-ray structures (PDF)

## Accession Codes

Coordinates and structure factors of the NfCYP51–analogue complexes are available in the Protein Data Bank (PDB) under accession codes 7RKR, 7RKT, and 7RKW. Authors will release the atomic coordinates and experimental data upon article publication.

## ABBREVIATIONS

BBB: blood–brain barrier
CNS: central nervous system
PAM: primary amoebic meningoencephalitis
MPO: multiparameter optimization
CDC: Centers for Disease Control and Prevention
AmpB: amphotericin B
NfCYP51: *N. fowleri* CYP51
DMF: dimethylformamide
DCM: dichloromethane
MeOH: methanol
e.e.: enantiomeric excess
SAR: structure–activity relationship
PK: pharmacokinetic
