# Supplementary material for: Miconazole-like Scaffold is a Promising Lead for Naegleria fowleri-Specific CYP51 Inhibitors
Source: J Med Chem. 2023 Dec 12;66(24):17059–73. doi: 10.1021/acs.jmedchem.3c01898 (PMC10758121; doi:10.1021/acs.jmedchem.3c01898)
Supplement: Supplementary file 2 — jm3c01898_si_002.pdf [file jm3c01898_si_002.pdf]

SUPPORTING INFORMATION

Miconazole-like Scaffold is a Promising Lead for *Naegleria fowleri*-Specific CYP51 Inhibitors

Vandna Sharma,[a,^] Valentina Noemi Madia,[b,^] Valeria Tudino,[c] Jennifer V. Nguyen,[a] Anjan Debnath,[a] Antonella Messore,[b] Davide Ialongo,[b] Elisa Patacchini,[b] Irene Palenca,[d] Silvia Basili Franzin,[d] Luisa Seguella,[d] Giuseppe Esposito,[d] Rita Petrucci,[e] Paola Di Matteo,[e] Martina Bortolami,[e] Francesco Saccoliti,[f] Roberto Di Santo,[b] Luigi Scipione,[b,] Roberta Costi,[b,⊥] and Larissa M. Podust[a,*]*

[a] Skaggs School of Pharmacy and Pharmaceutical Sciences, Center for Discovery and Innovation in Parasitic Diseases, University of California San Diego, La Jolla, CA 92093, USA

[b] Dipartimento di Chimica e Tecnologie del Farmaco, Istituto Pasteur-Fondazione Cenci Bolognetti, "Sapienza" Università di Roma, p.le Aldo Moro 5, I-00185, Rome, Italy

[c] Università degli Studi di Siena, Dipartimento di Biotecnologie, Chimica e Farmacia via Aldo Moro 2, 53100, Siena, Italy

[d] Department of Physiology and Pharmacology "V. Erspamer", "Sapienza" Università di Roma, p.le Aldo Moro 5, I-00185, Rome, Italy

[e] Dipartimento di Scienze di Base e Applicate per l'Ingegneria, "Sapienza" Università di Roma, Via Castro Laurenziano 7, 00161, Rome, Italy

[f] D3 PharmaChemistry, Italian Institute of Technology, Via Morego 30, 16163, Genova, Italy

^These authors contributed equally

⊥Senior author

*Corresponding authors. E-mail: lpodust@health.ucsd.edu; luigi.scipione@uniroma1.it

Table of contents

Table S1 S3

Table S2 S4

^{1}H and ^{13}C NMR spectra of compounds **3a-5a, 3b-6b, 7, 8b-10b** S5

HPLC traces of lead compounds **8b** and **9b** S16

SMILES formulas for compounds **1, 2a**, **3a-5a, 3b-6b, 7, 8b-10b, 11-18** and related data is provided in a separate CSV file

Table S1. Hits with ≥50% inhibition at 10 μM.

Compound	Structure	MW (g/mol)	% Inhibition at 10 μM*
**2a**	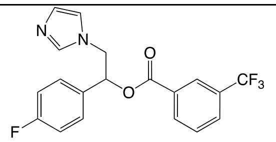	379.33	51.5 ± 13.4
**2b**	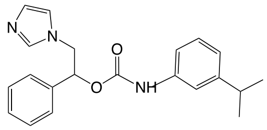	349.43	63.0 ± 9.9
**2c**	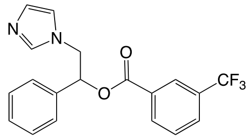	360.33	53.5 ± 6.4
**2d**	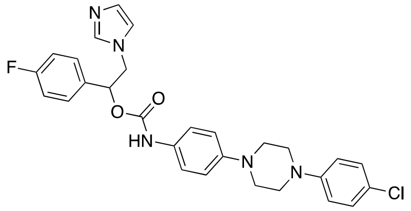	504.9	66.5 ± 3.5
**2e**	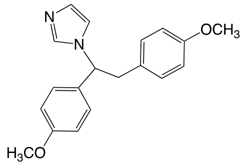	308.38	49.5 ± 6.4
**2f**	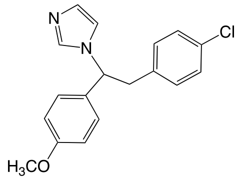	312.80	54.5 ± 0.7
**2g**	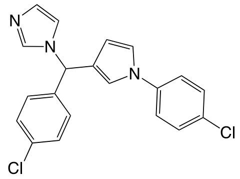	368.26	47.0 ± 7.1
**2h**	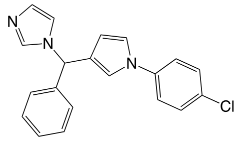	333.82	49.0 ± 9.9
**2i**	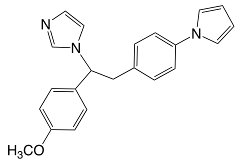	343.43	37.5 ± 30.4

*Mean of two biological duplicates.

Table S2. Retention time (t_R), dwell time, MRM transition, linear relation (R^2) and matrix effect (ME) in both biological matrix (brain B and plasma P) of **9b**, **8b**, and **1** reported according to the elution order.

Compound	t_R (min)	dwell time (s)	MRM (*m/z*)	R^2	ME (%) in B	ME (%) in P
**9b**	6.66	0.200	365 > 159	0.9994	-7.75	-20.31
**8b**	8.89	0.200	415 > 159	0.9942	18.34	0.15
**1**	10.30	0.200	417 > 159<br>417 > 161	0.9980	15.02	8.23

^{1}H and ^{13}C NMR spectra of compounds **3a-5a, 3b-6b, 7, 8b-10b**

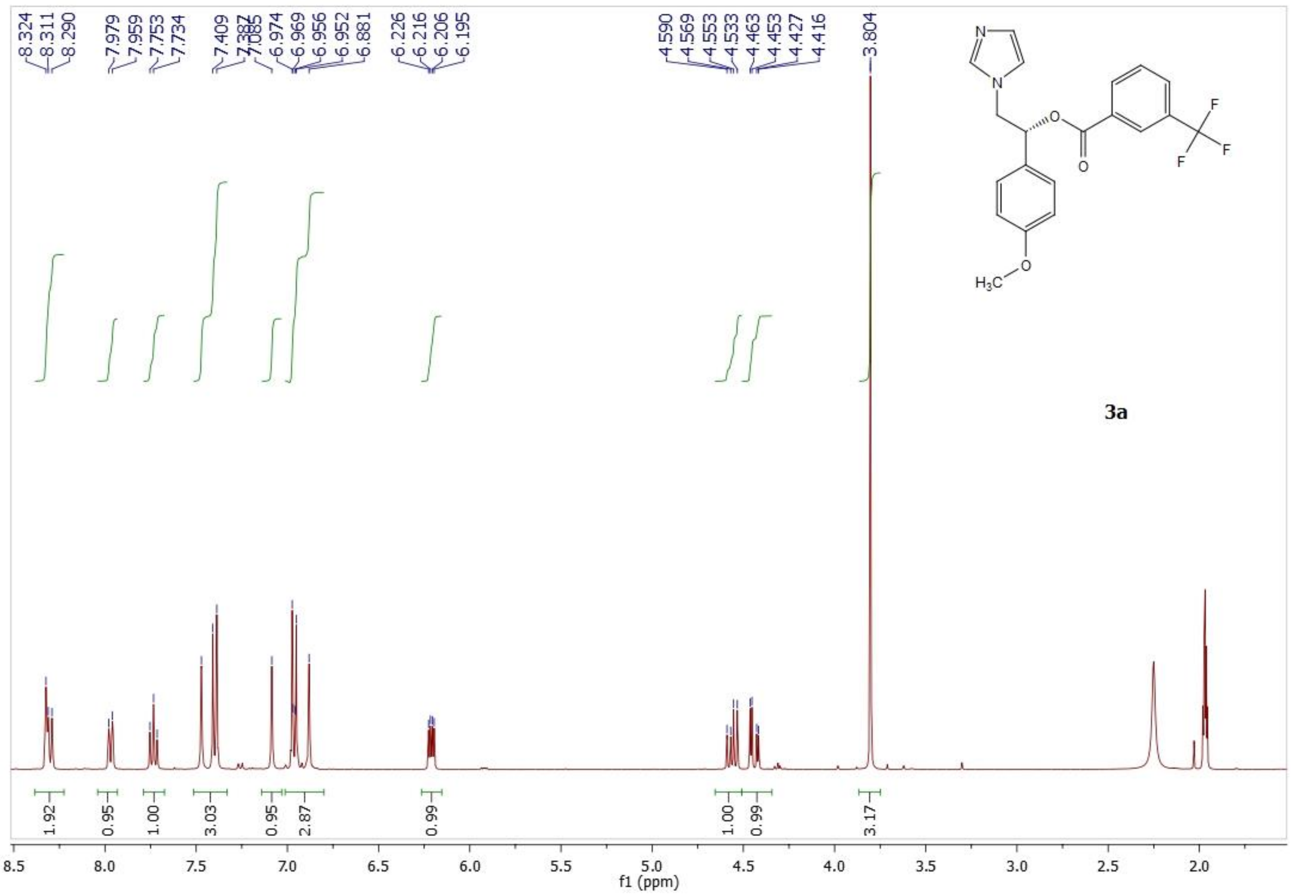

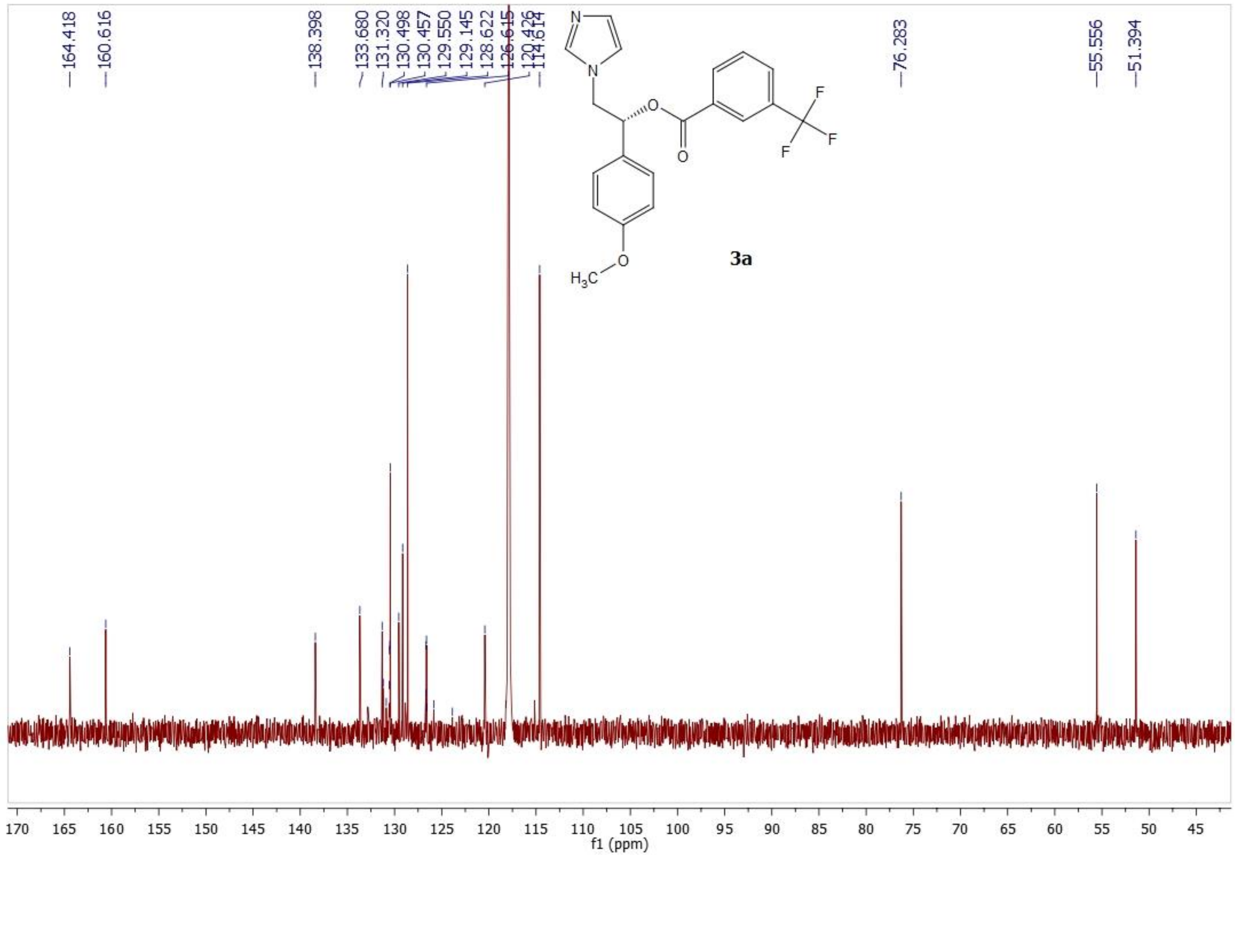

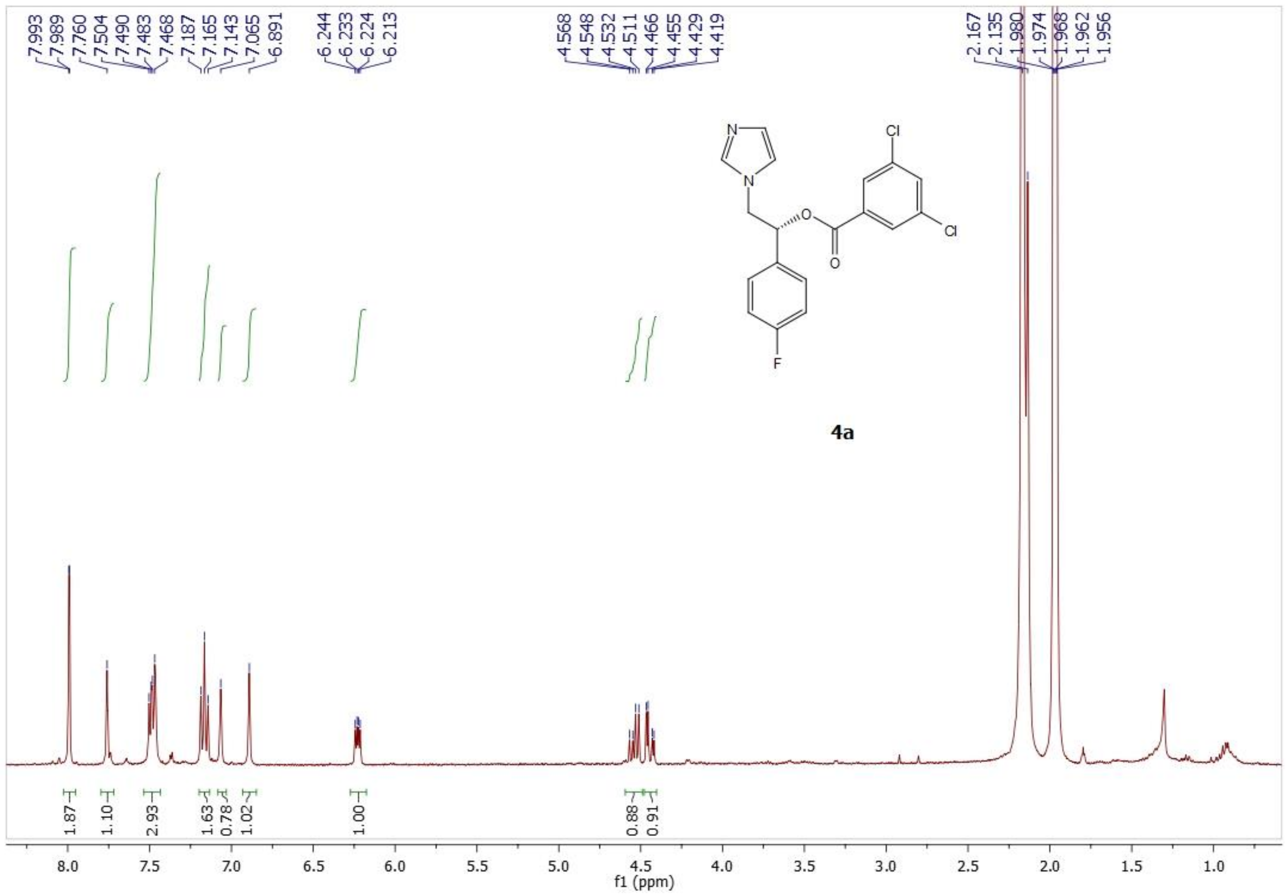
7.993
7.989
7.760
7.504
7.490
7.483
7.468
7.187
7.165
7.143
7.065
6.891
6.244
6.233
6.224
6.213
4.568
4.548
4.532
4.511
4.466
4.455
4.429
4.419
2.167
2.135
1.980
1.974
1.968
1.962
1.956
N
Cl
O
Cl
O
N
F
4a
1.87
1.10
2.93
1.63
0.78
1.02
1.00
0.88
0.91
f1 (ppm)

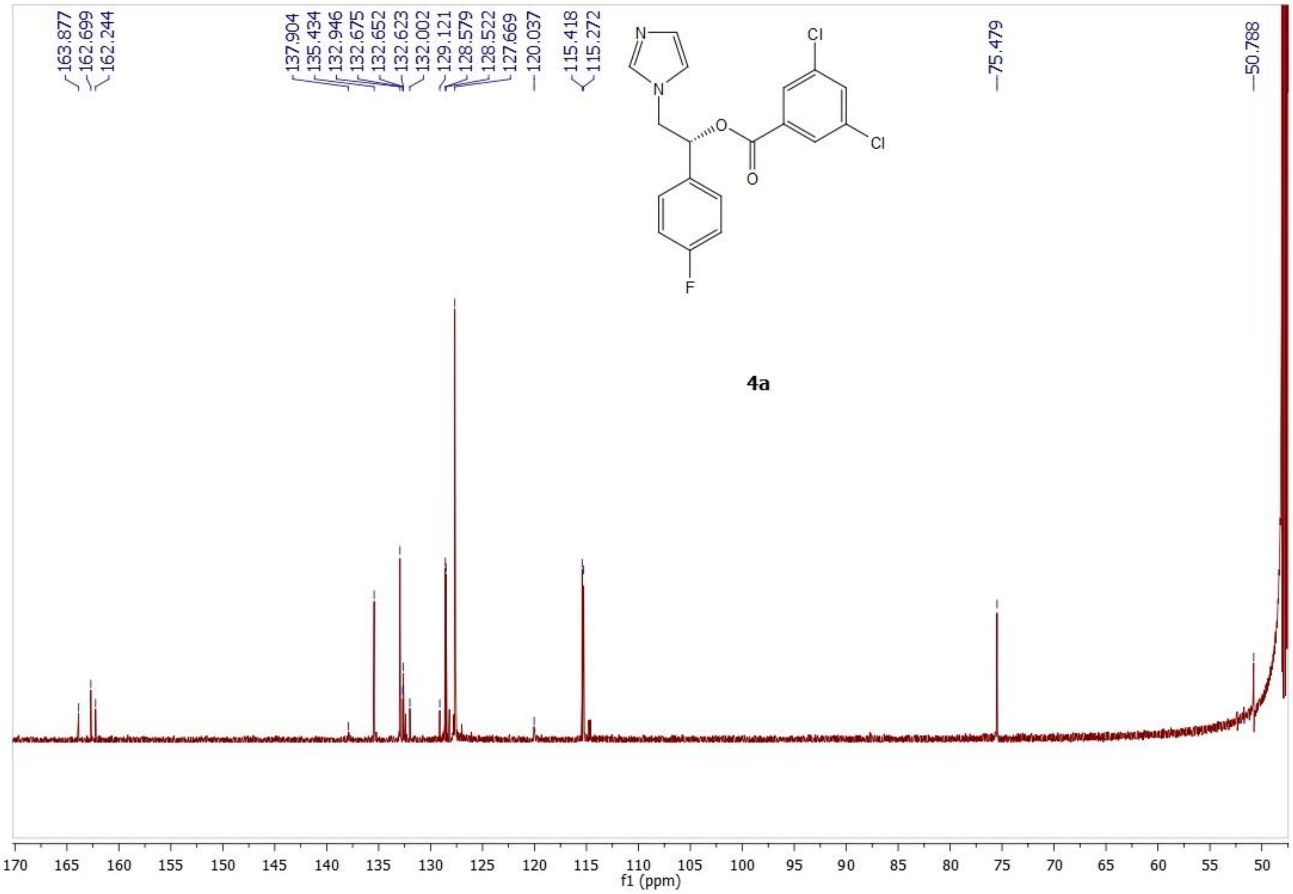
163.877
162.699
162.244
137.904
135.434
132.946
132.675
132.652
132.623
132.002
129.121
128.579
128.522
127.669
120.037
115.418
115.272
75.479
50.788
N
Cl
O
Cl
O
N
F
4a
f1 (ppm)

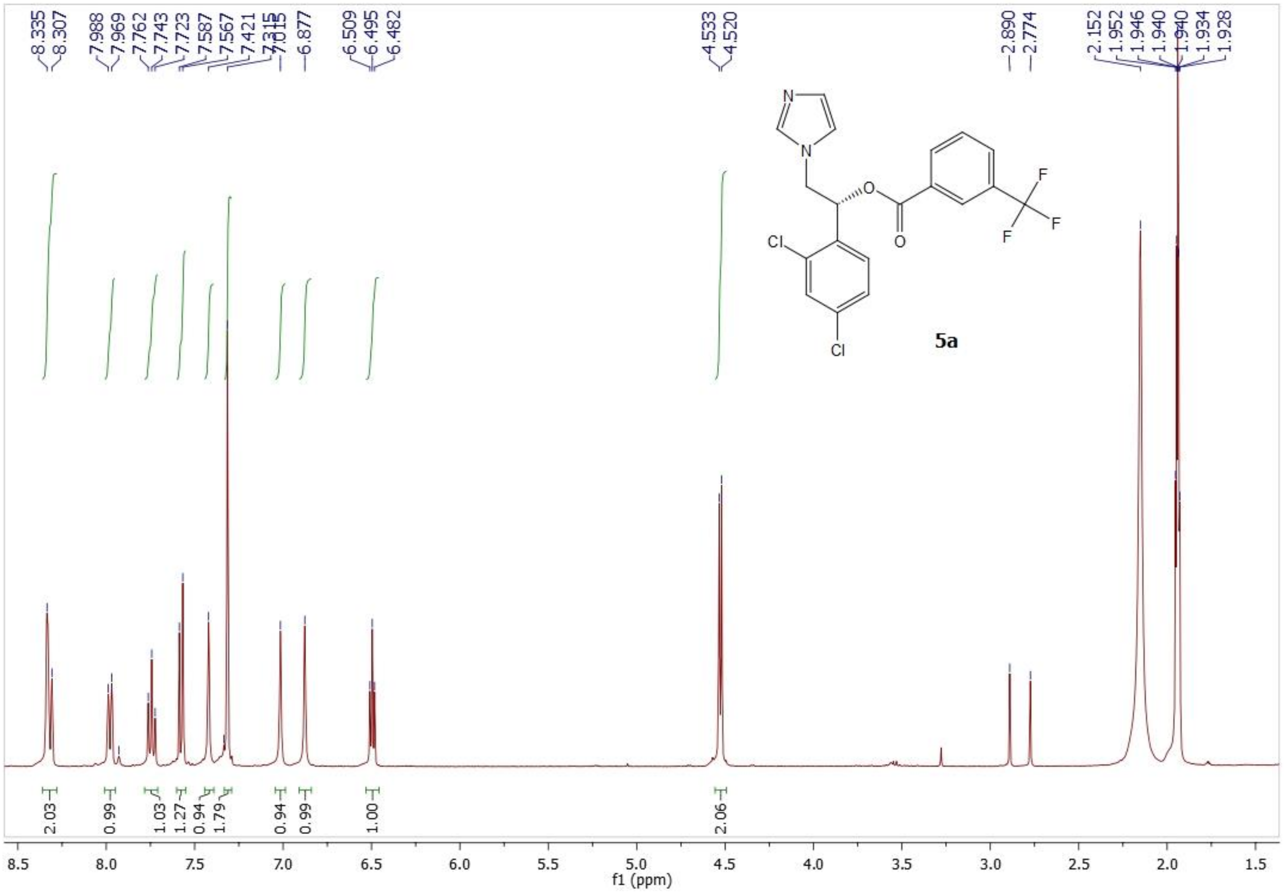
8.335
8.307
7.988
7.969
7.762
7.743
7.723
7.587
7.567
7.421
7.315
7.015
6.877
6.509
6.495
6.482
4.533
4.520
2.890
2.774
2.152
1.952
1.946
1.940
1.940
1.934
1.928
5a
2.03
0.99
1.03
1.27
0.94
1.79
0.94
0.99
1.00
2.06
f1 (ppm)

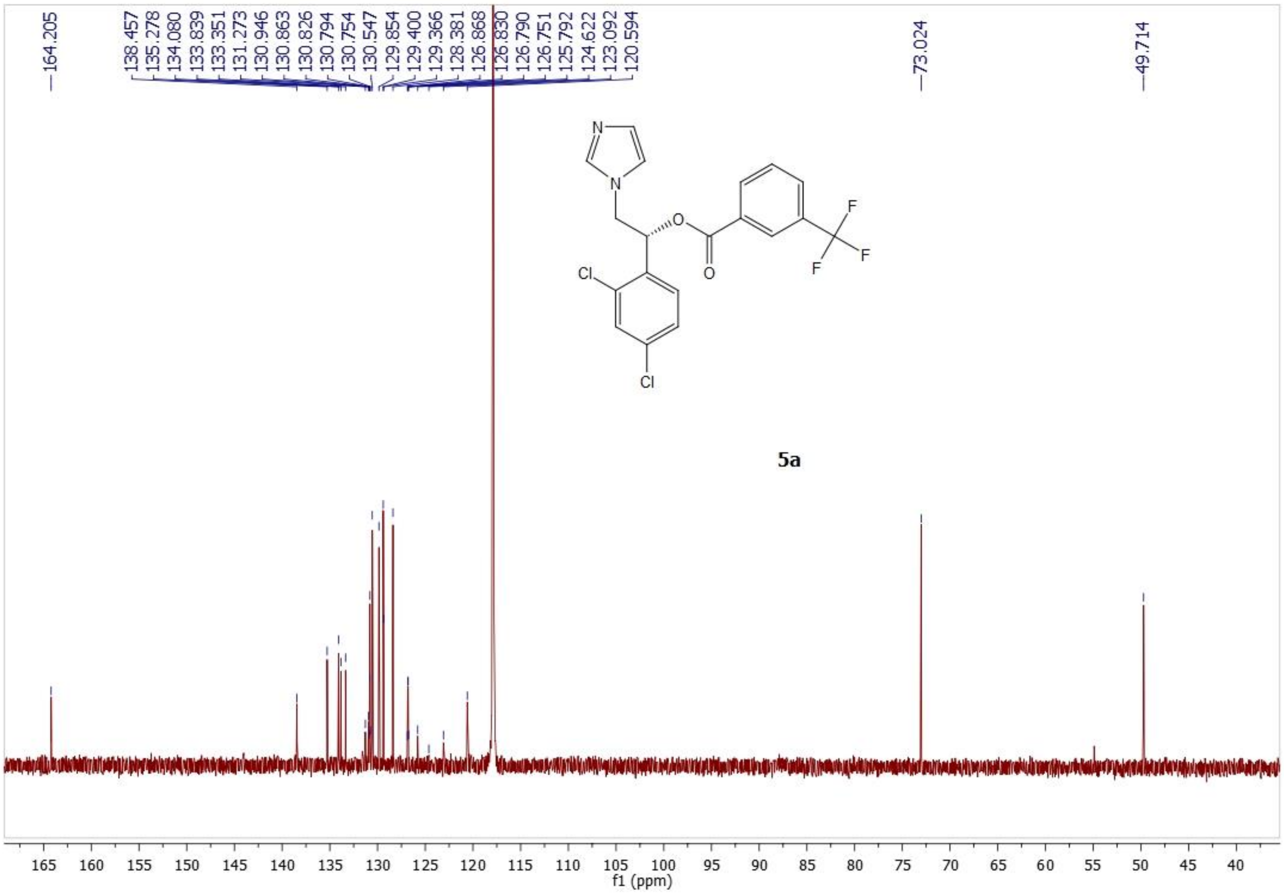
164.205
138.457
135.278
134.080
133.839
133.351
131.273
130.946
130.863
130.826
130.794
130.754
130.547
129.854
129.400
129.366
128.381
126.868
126.830
126.790
126.751
125.792
124.622
123.092
120.594
73.024
49.714
5a
f1 (ppm)

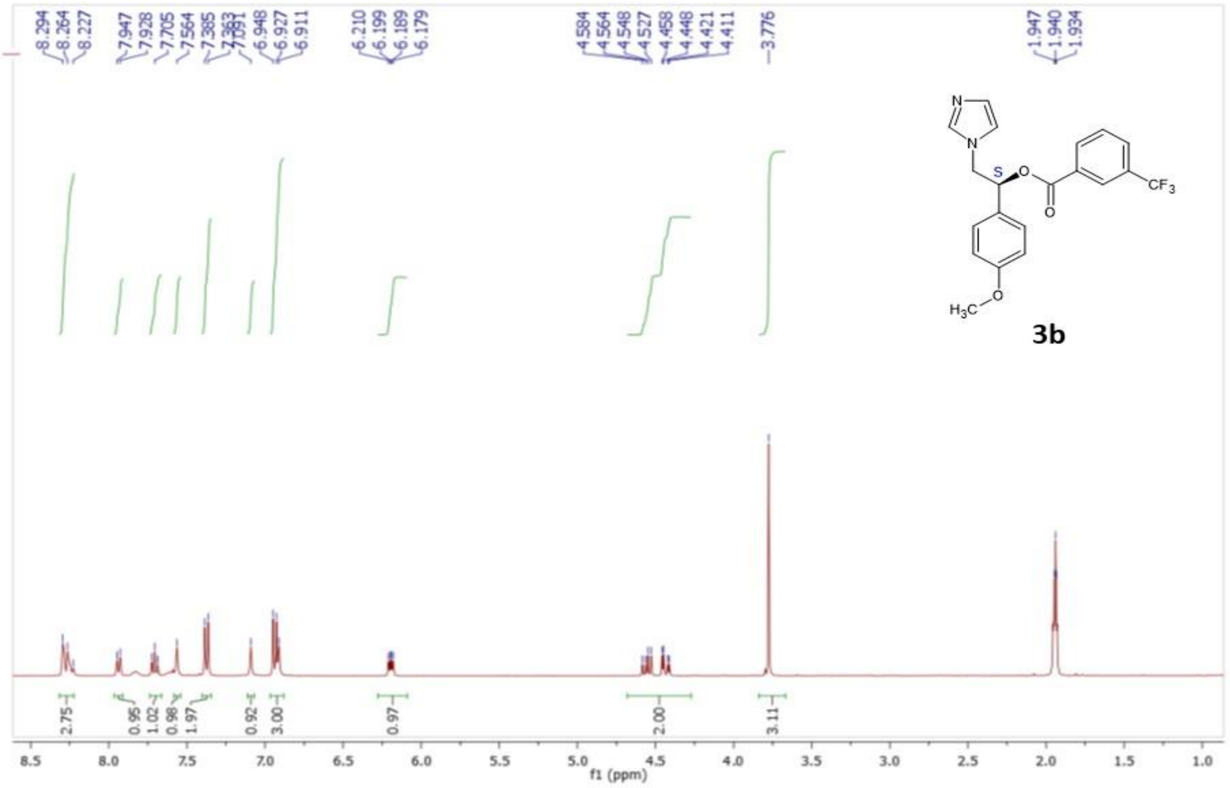
3b
f1 (ppm)

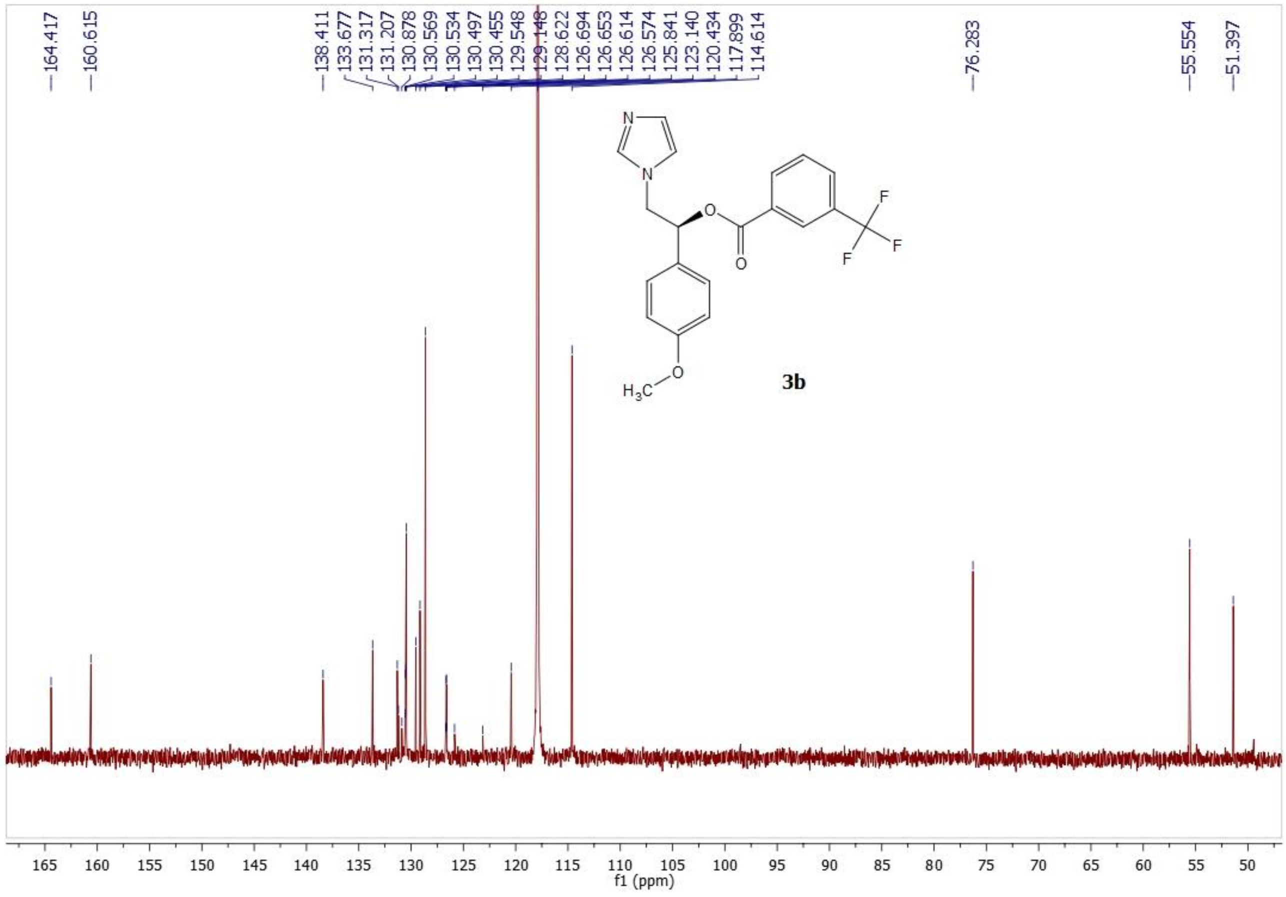
3b
f1 (ppm)

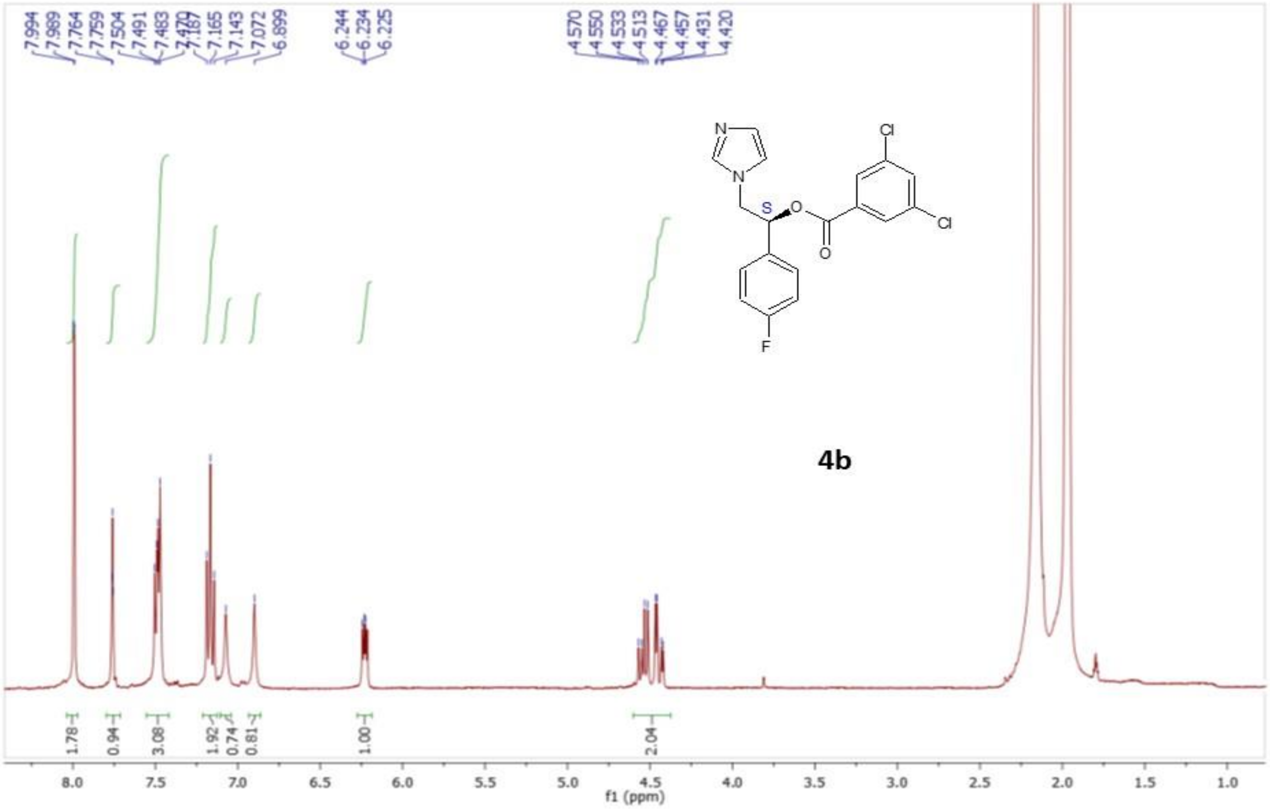
4b
f1 (ppm)

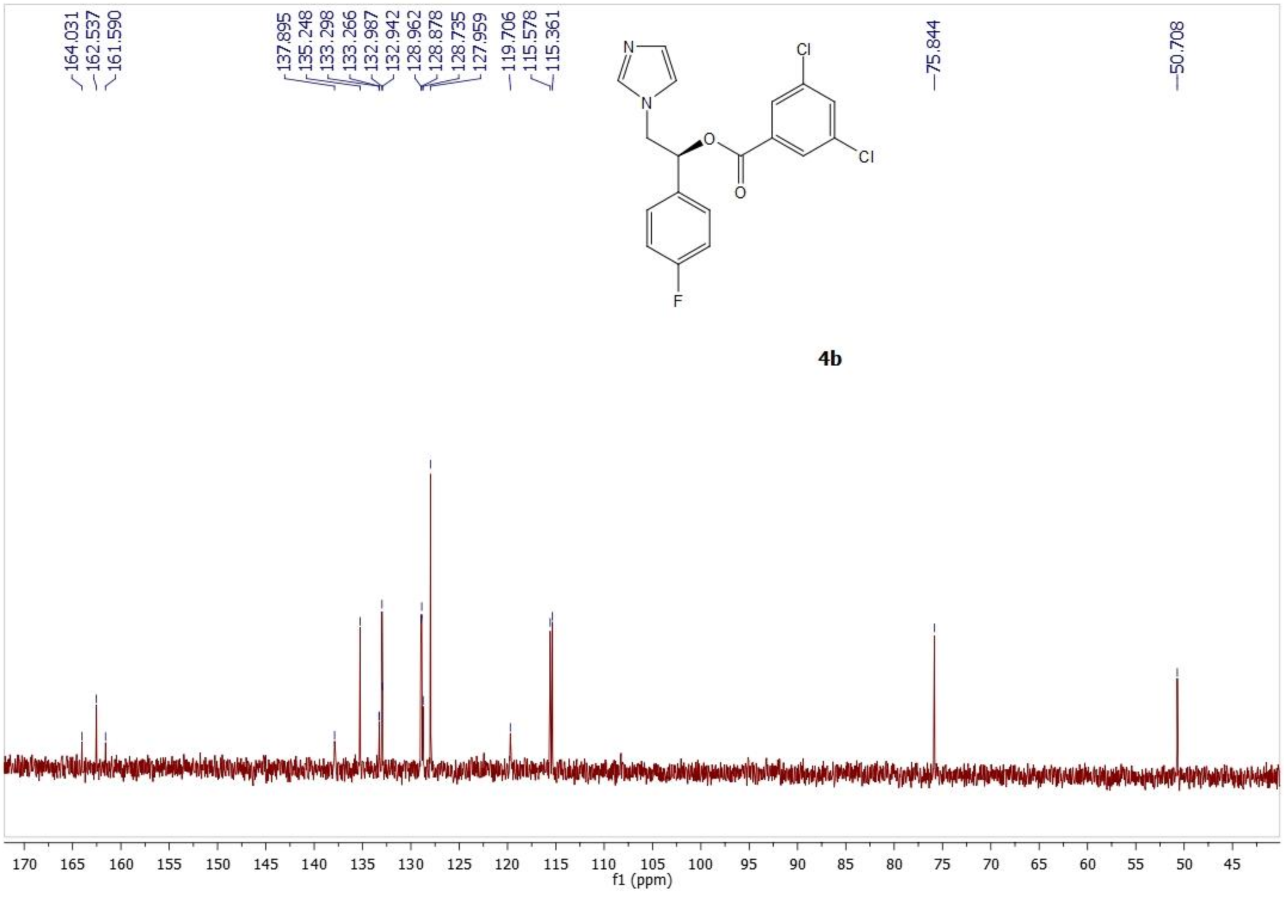
4b
f1 (ppm)

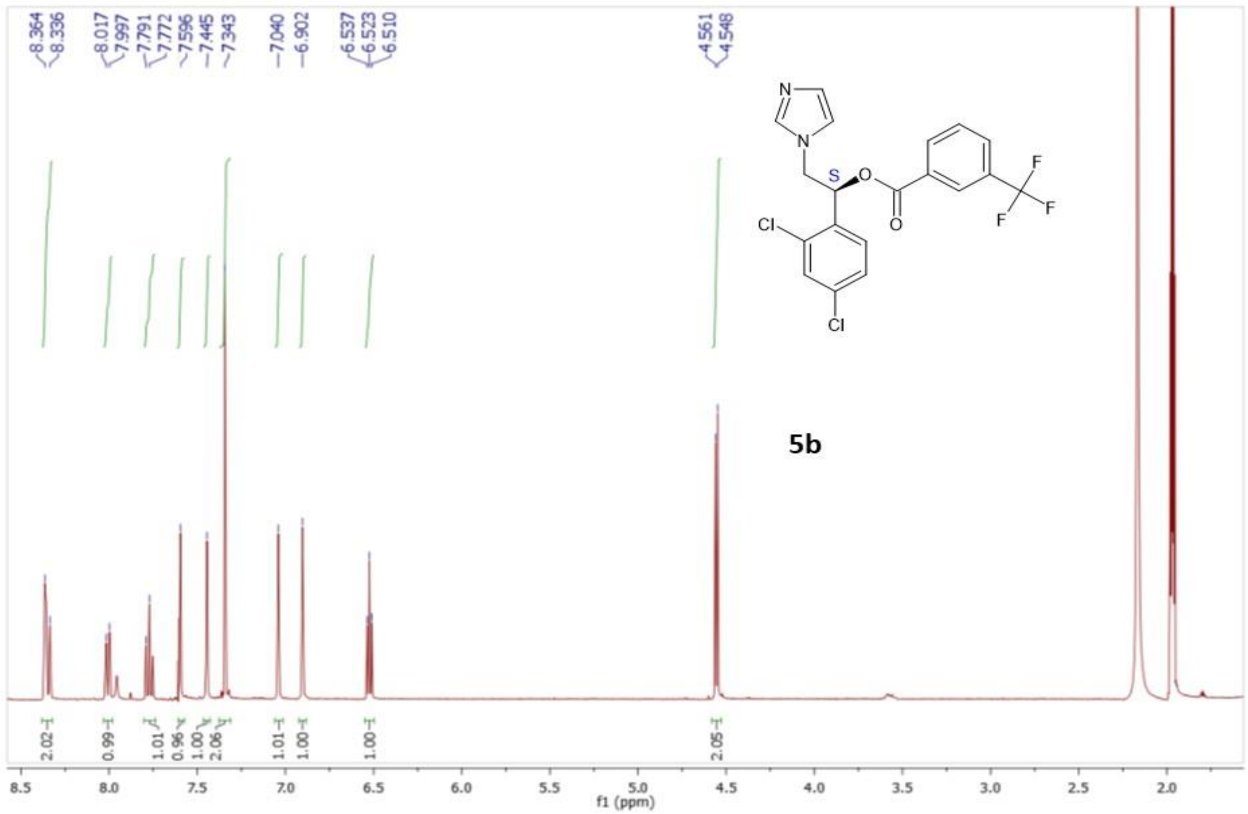
8.364
8.336
8.017
7.997
7.791
7.772
7.596
7.445
7.343
7.040
6.902
6.537
6.523
6.510
4.561
4.548
5b
2.02
0.99
1.01
0.96
1.00
2.06
1.01
1.00
1.00
2.05
f1 (ppm)

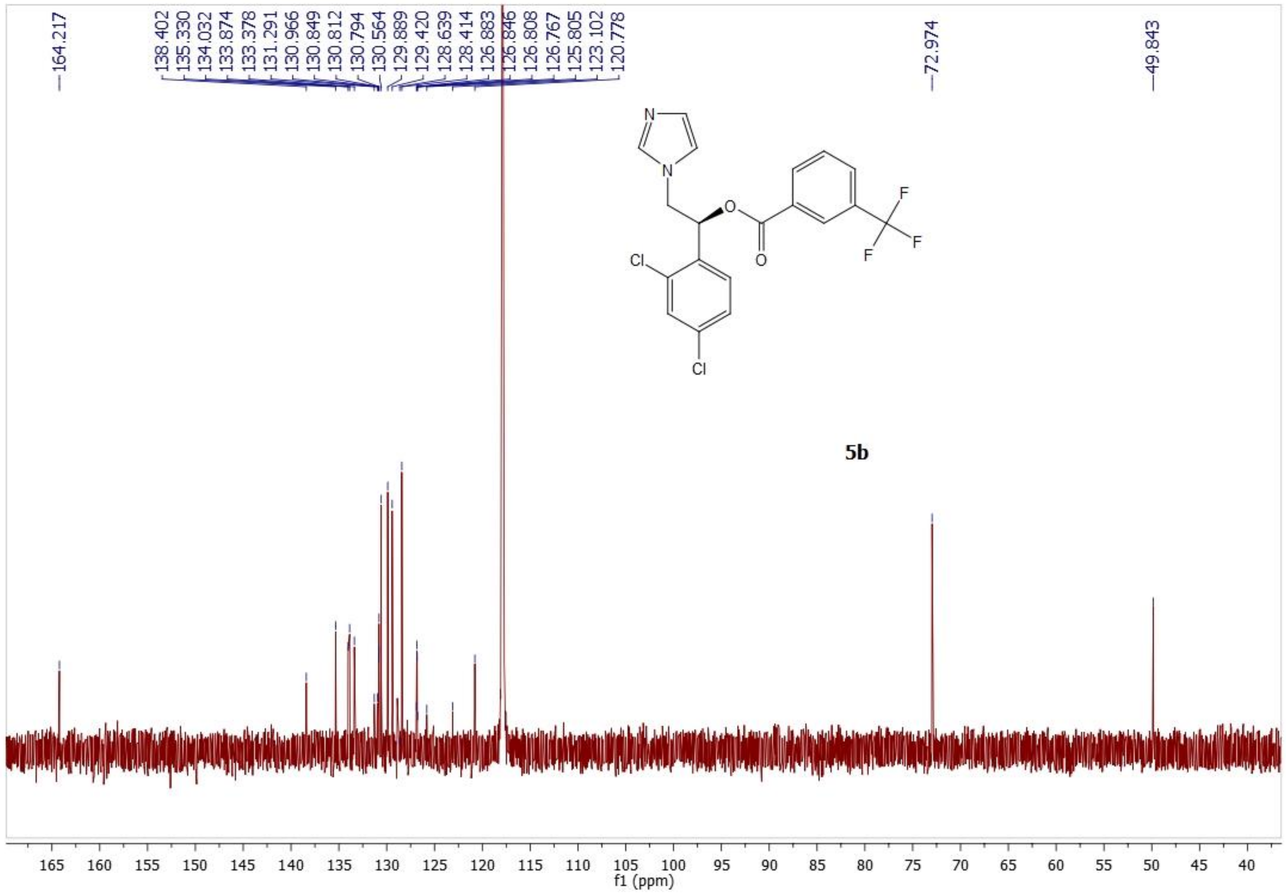
164.217
138.402
135.330
134.032
133.874
133.378
131.291
130.966
130.849
130.812
130.794
130.564
129.889
129.420
128.639
128.414
126.883
126.846
126.808
126.767
125.805
123.102
120.778
72.974
49.843
5b
f1 (ppm)

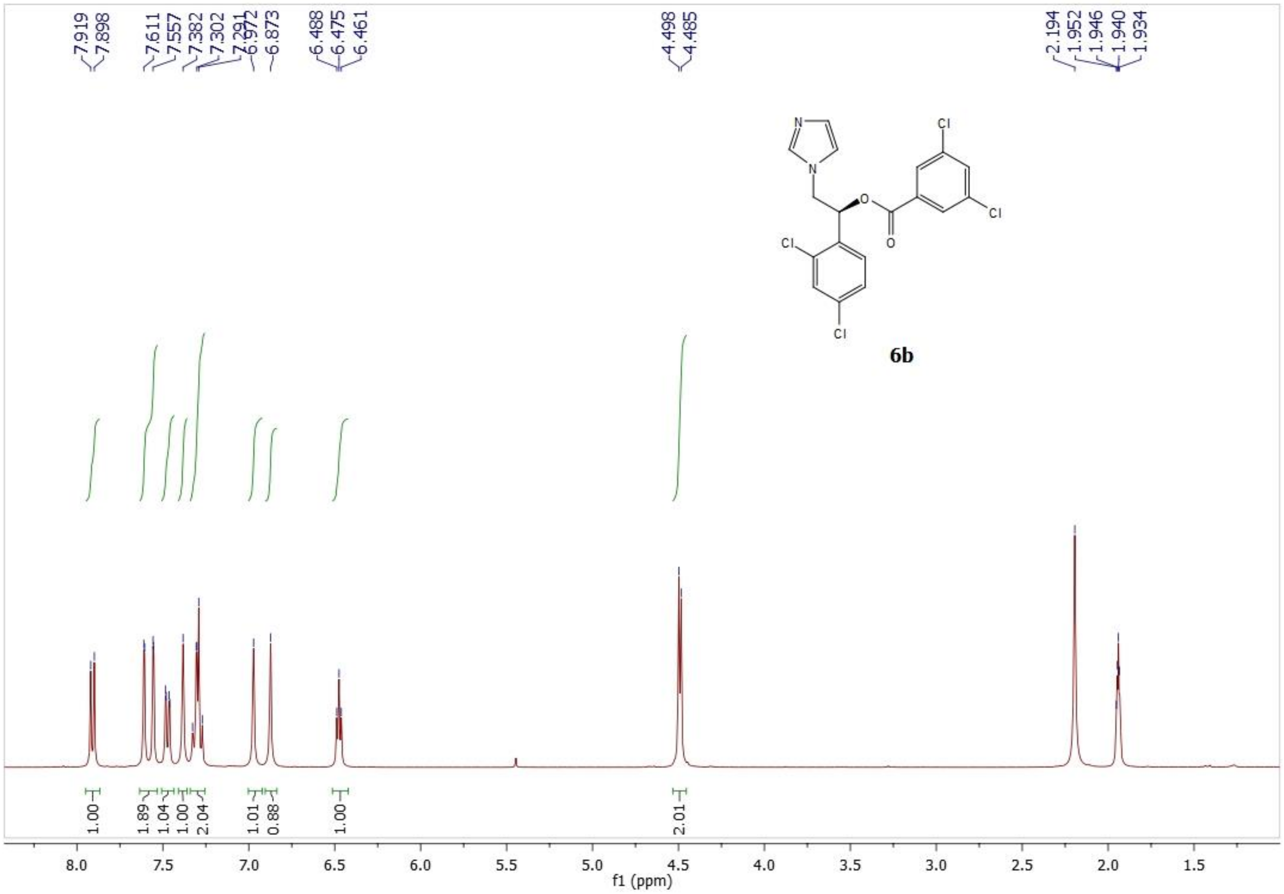
7.919
7.898
7.611
7.557
7.382
7.302
7.291
6.972
6.873
6.488
6.475
6.461
4.498
4.485
2.194
1.952
1.946
1.940
1.934
6b
1.00
1.89
1.04
1.00
2.04
1.01
0.88
1.00
2.01
f1 (ppm)

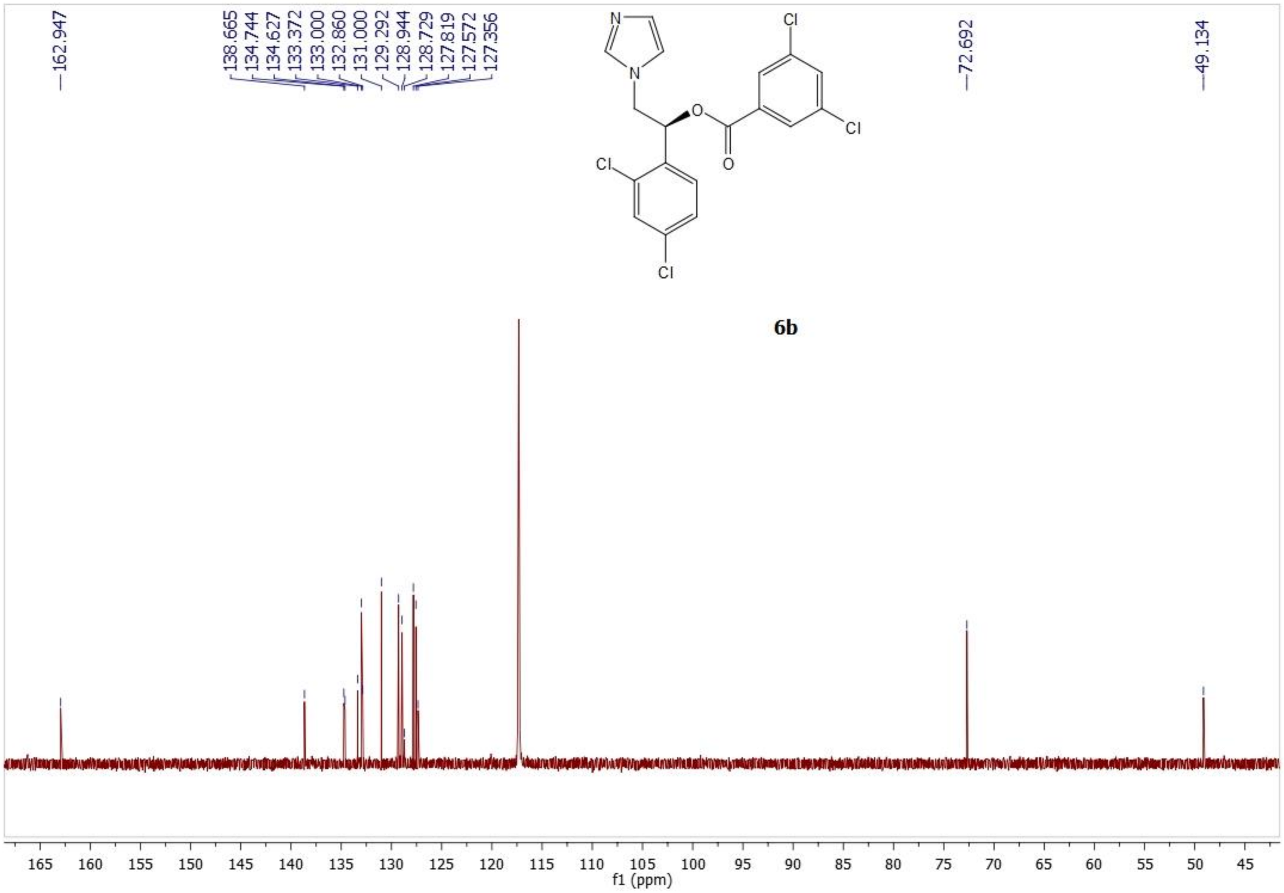
162.947
138.665
134.744
134.627
133.372
133.000
132.860
131.000
129.292
128.944
128.729
127.819
127.572
127.356
72.692
49.134
6b
f1 (ppm)

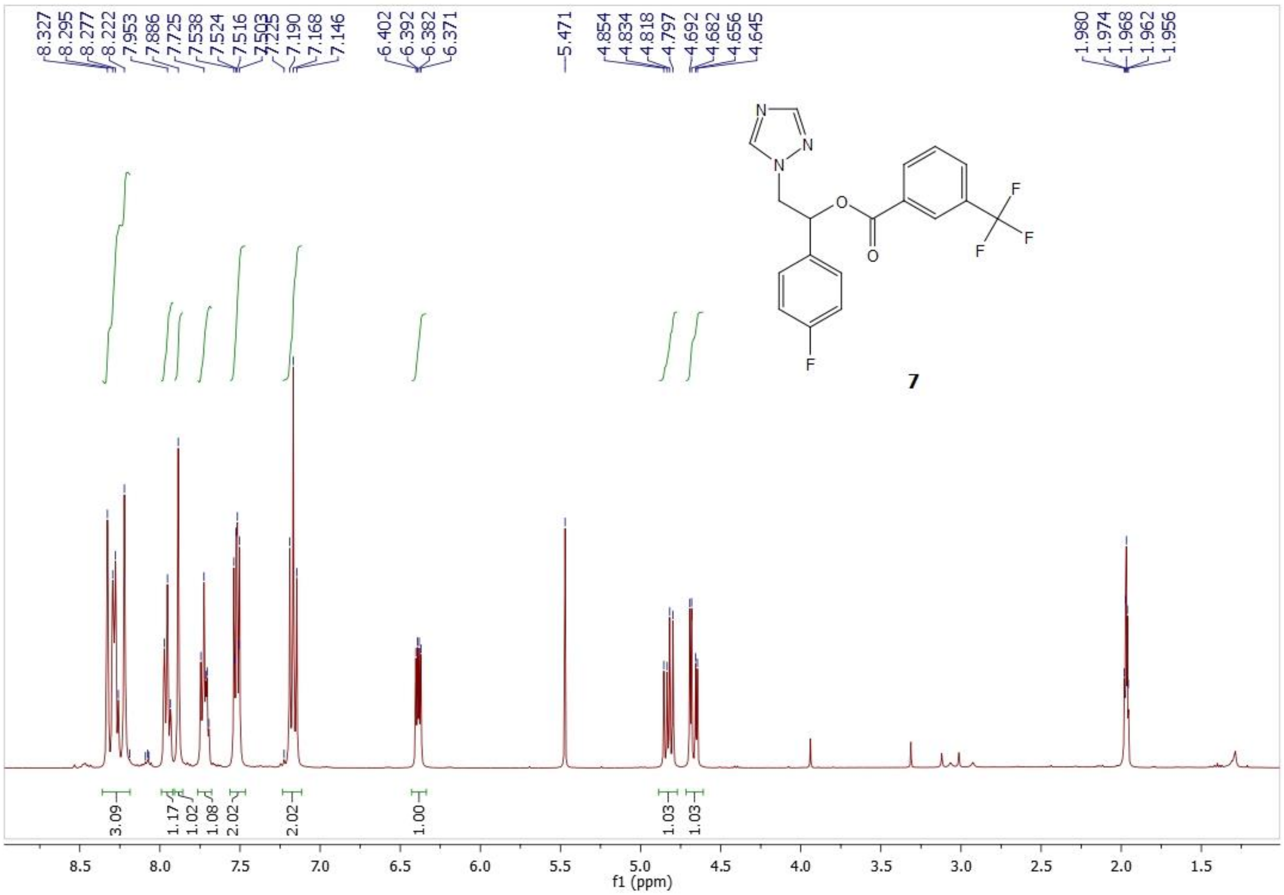
8.327
8.295
8.277
8.222
7.953
7.886
7.725
7.538
7.524
7.516
7.503
7.225
7.190
7.168
7.146
6.402
6.392
6.382
6.371
5.471
4.854
4.834
4.818
4.797
4.692
4.682
4.656
4.645
1.980
1.974
1.968
1.962
1.956
N
N
N
O
O
F
F
F
F
7
3.09
1.17
1.02
1.08
2.02
2.02
1.00
1.03
1.03
f1 (ppm)

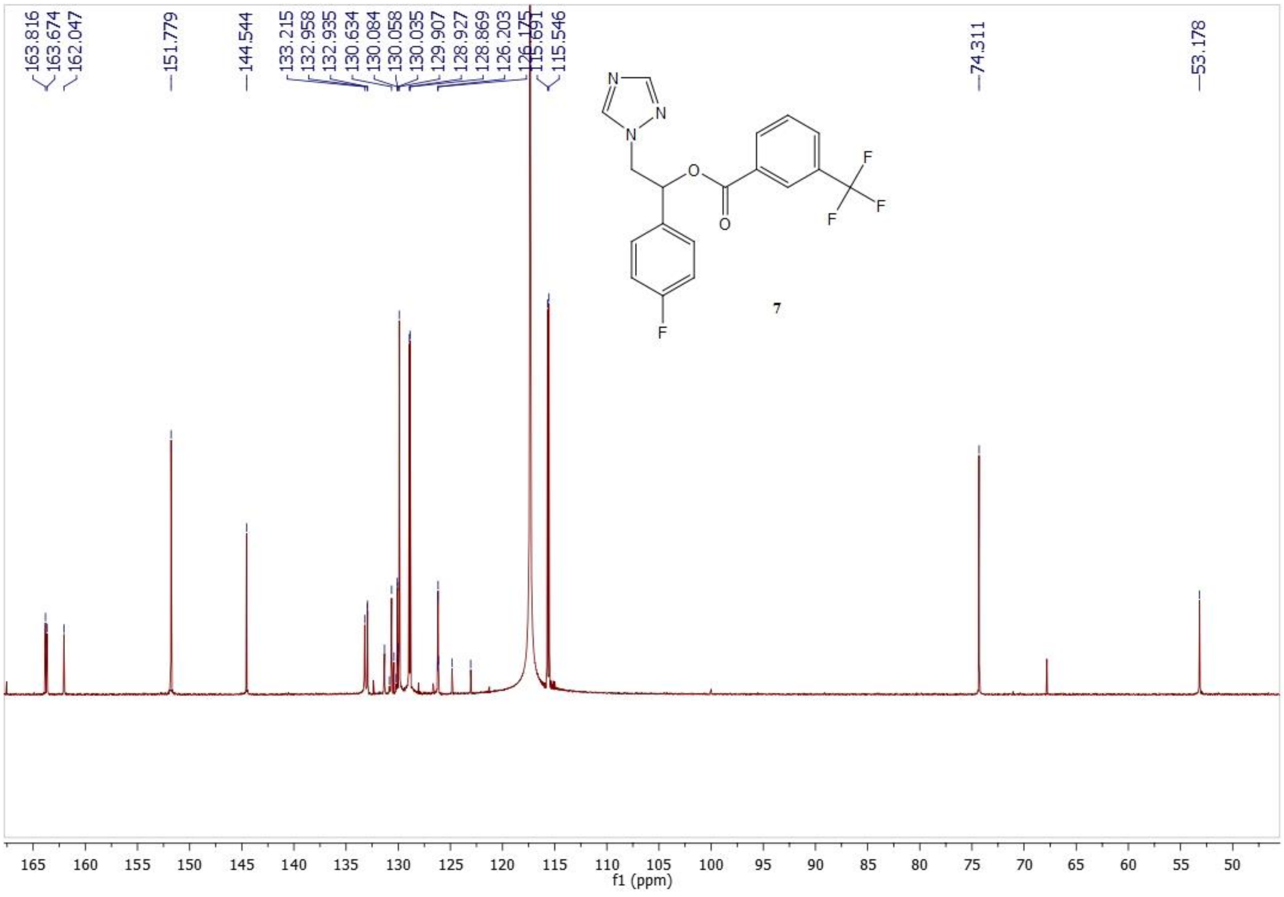
163.816
163.674
162.047
151.779
144.544
133.215
132.958
132.935
130.634
130.084
130.058
130.035
129.907
128.927
128.869
126.203
126.175
115.691
115.546
74.311
53.178
N
N
N
O
O
F
F
F
F
7
f1 (ppm)

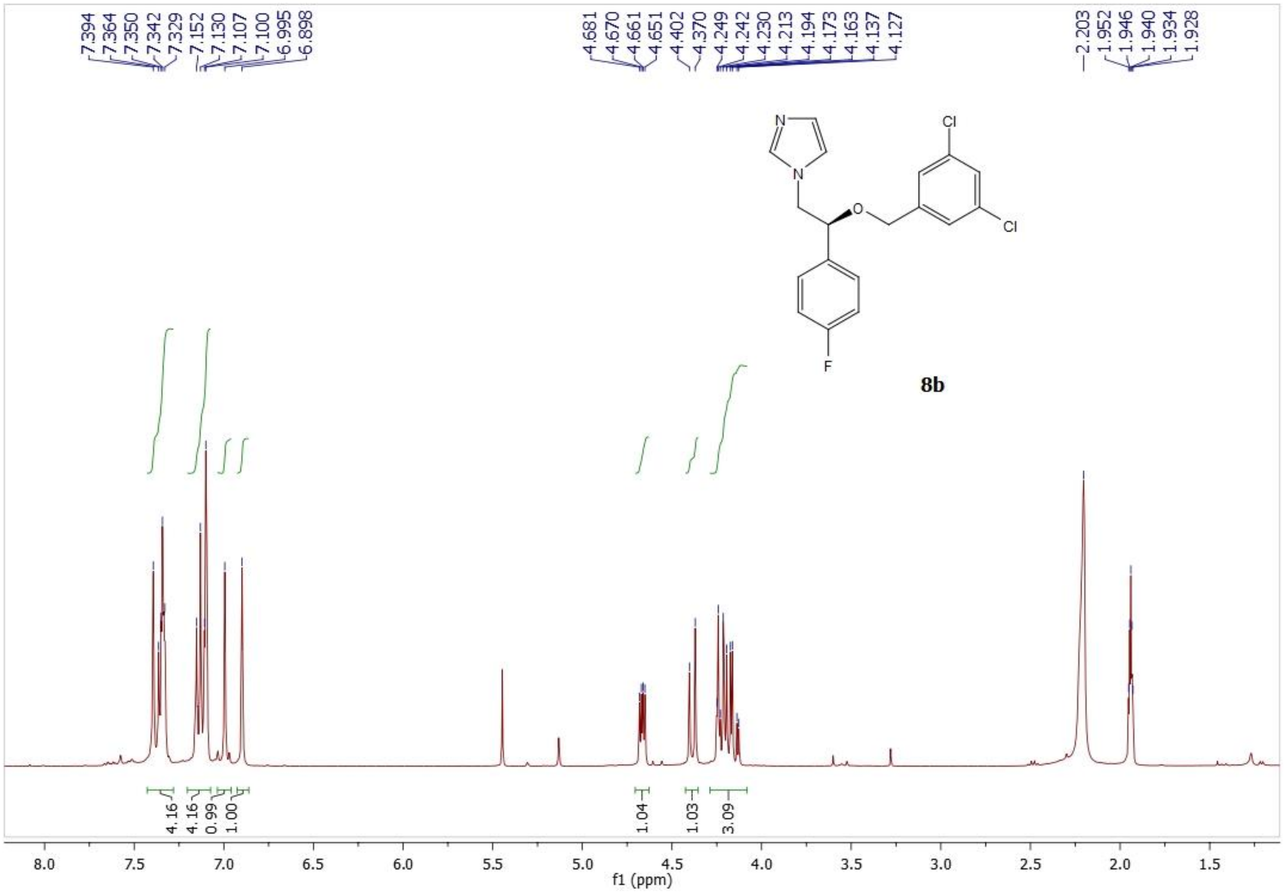
7.394
7.364
7.350
7.342
7.329
7.152
7.130
7.107
7.100
6.995
6.898
4.681
4.670
4.661
4.651
4.402
4.370
4.249
4.242
4.230
4.213
4.194
4.173
4.163
4.137
4.127
2.203
1.952
1.946
1.940
1.934
1.928
N
N
O
Cl
Cl
F
8b
4.16
4.16
0.99
1.00
1.04
1.03
3.09
8.0 7.5 7.0 6.5 6.0 5.5 5.0 4.5 4.0 3.5 3.0 2.5 2.0 1.5
f1 (ppm)

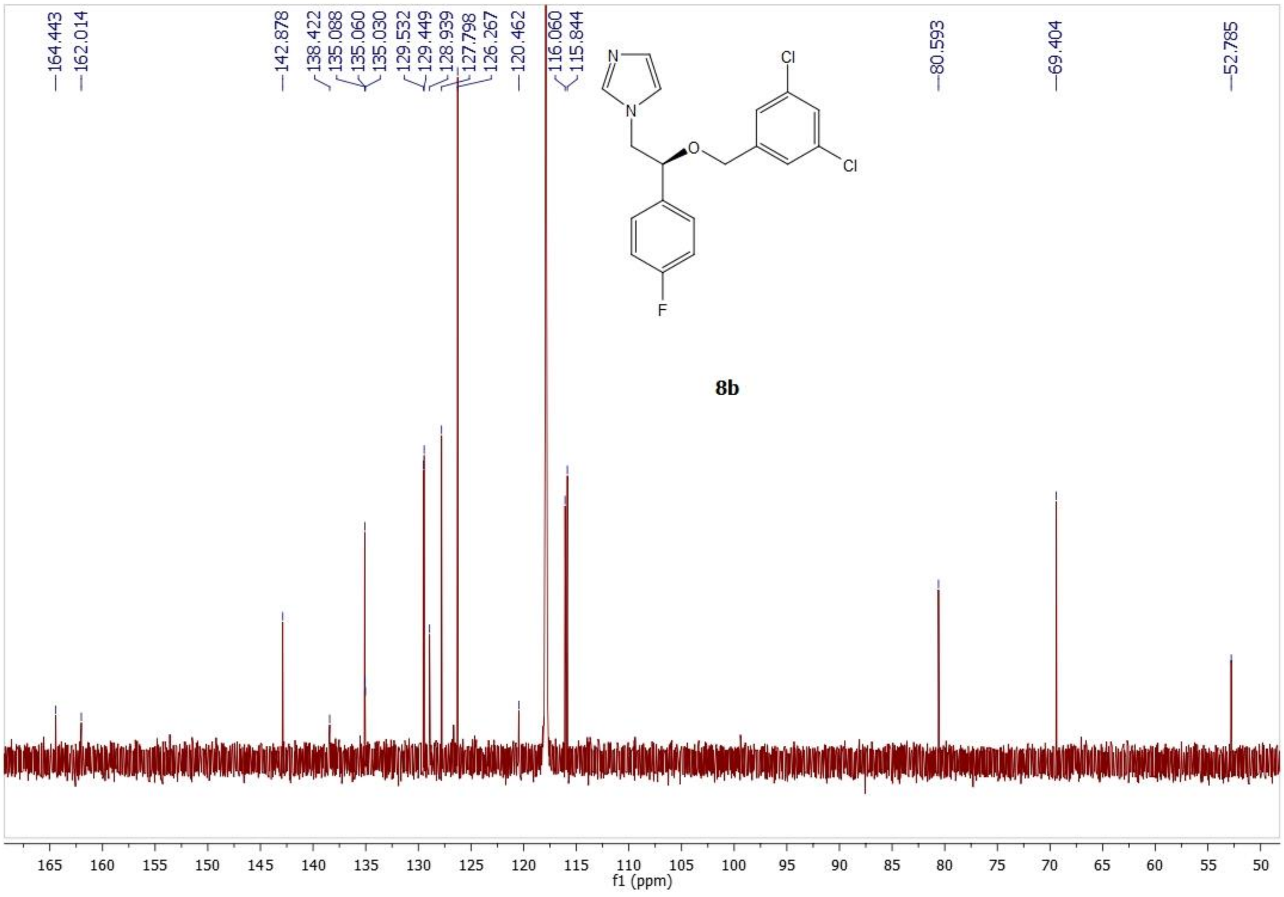
164.443
162.014
142.878
138.422
135.088
135.060
135.030
129.532
129.449
128.939
127.798
126.267
120.462
116.060
115.844
80.593
69.404
52.785
N
N
O
Cl
Cl
F
8b
165 160 155 150 145 140 135 130 125 120 115 110 105 100 95 90 85 80 75 70 65 60 55 50
f1 (ppm)

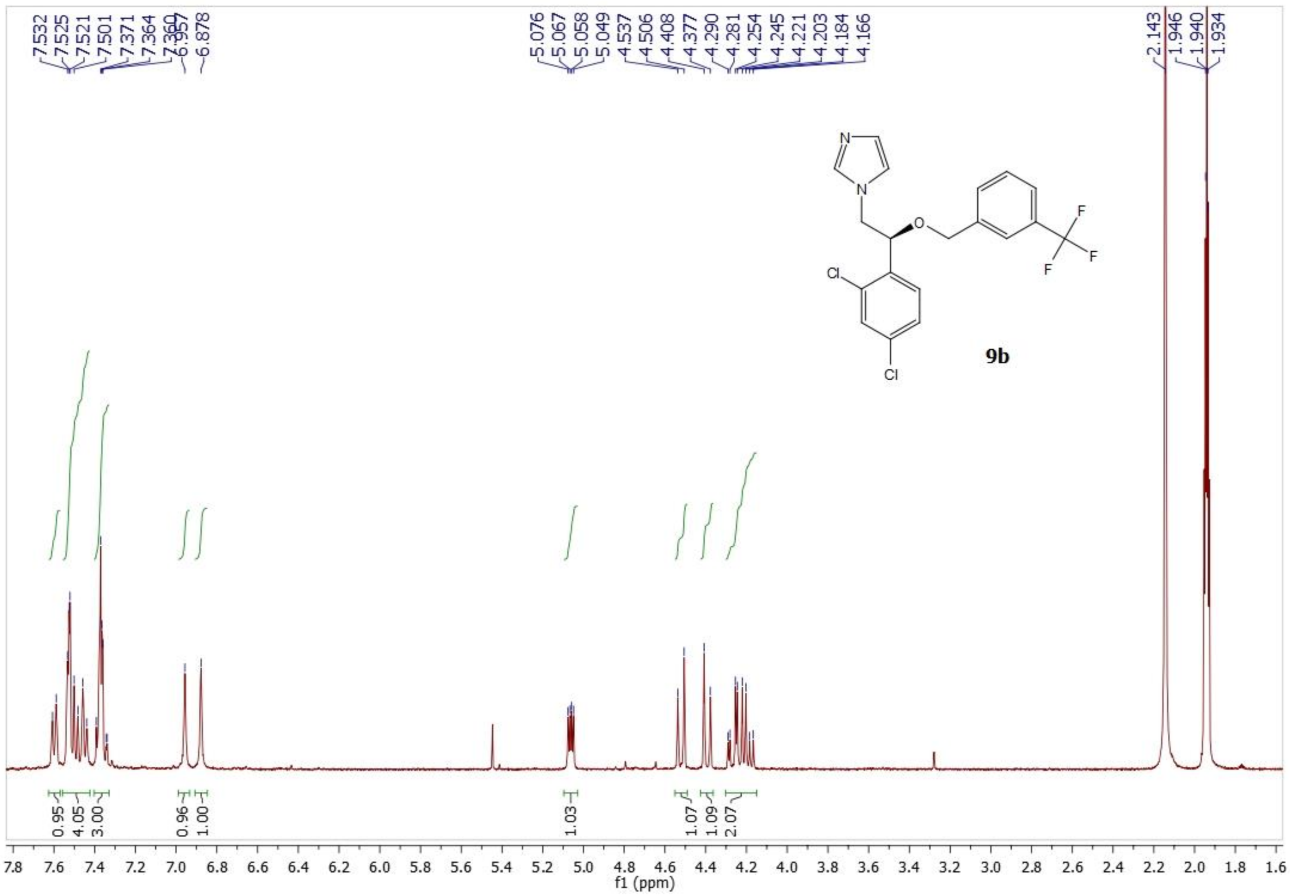
7.532
7.525
7.521
7.501
7.371
7.364
7.360
6.957
6.878
5.076
5.067
5.058
5.049
4.537
4.506
4.408
4.377
4.290
4.281
4.254
4.245
4.221
4.203
4.184
4.166
2.143
1.946
1.940
1.934
N
N
O
F
F
F
Cl
Cl
9b
0.95
4.05
3.00
0.96
1.00
1.03
1.07
1.09
2.07
f1 (ppm)

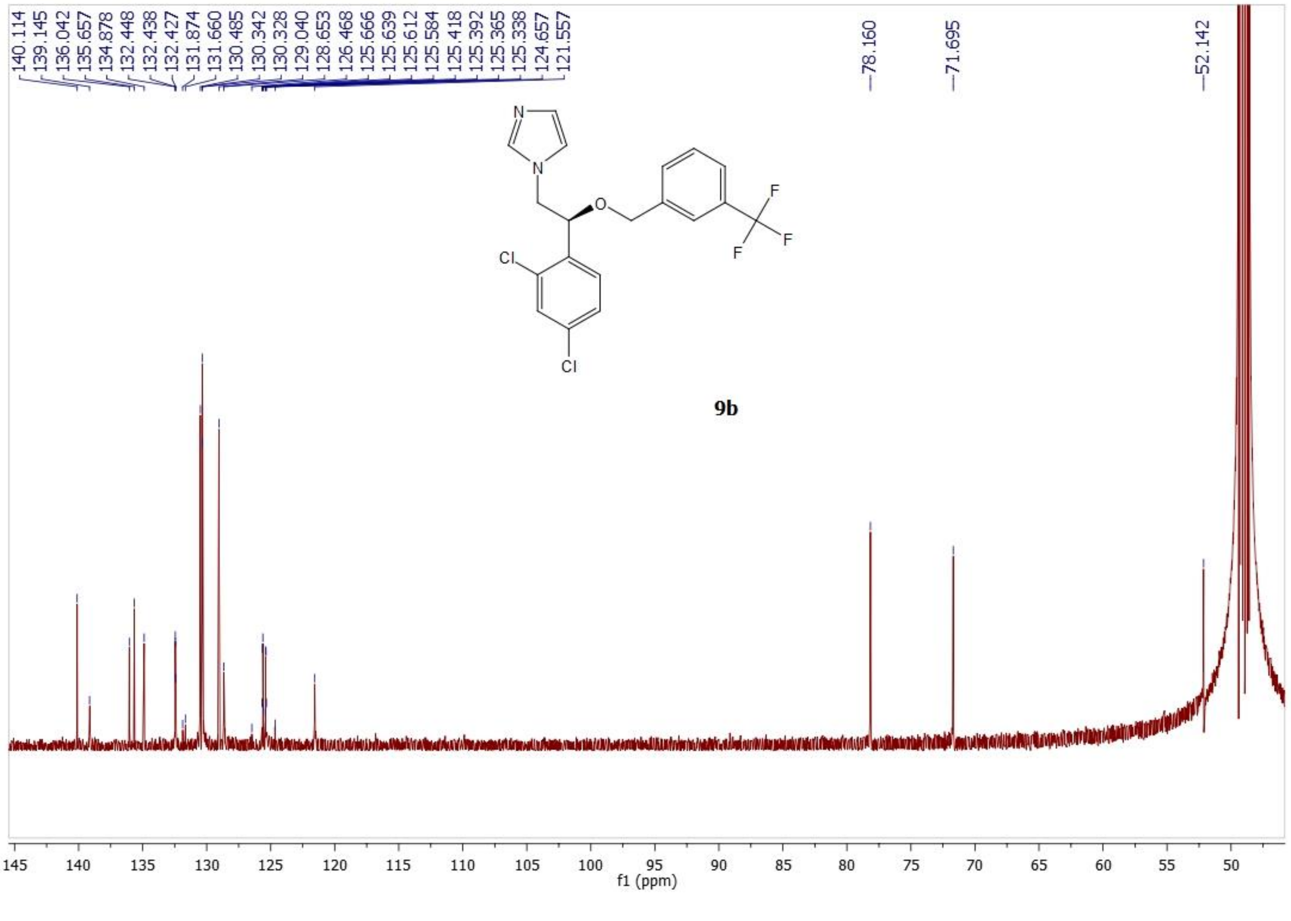
140.114
139.145
136.042
135.657
134.878
132.448
132.438
132.427
131.874
131.660
130.485
130.342
130.328
129.040
128.653
126.468
125.666
125.639
125.612
125.584
125.418
125.392
125.365
125.338
124.657
121.557
78.160
71.695
52.142
N
N
O
F
F
F
Cl
Cl
9b
f1 (ppm)

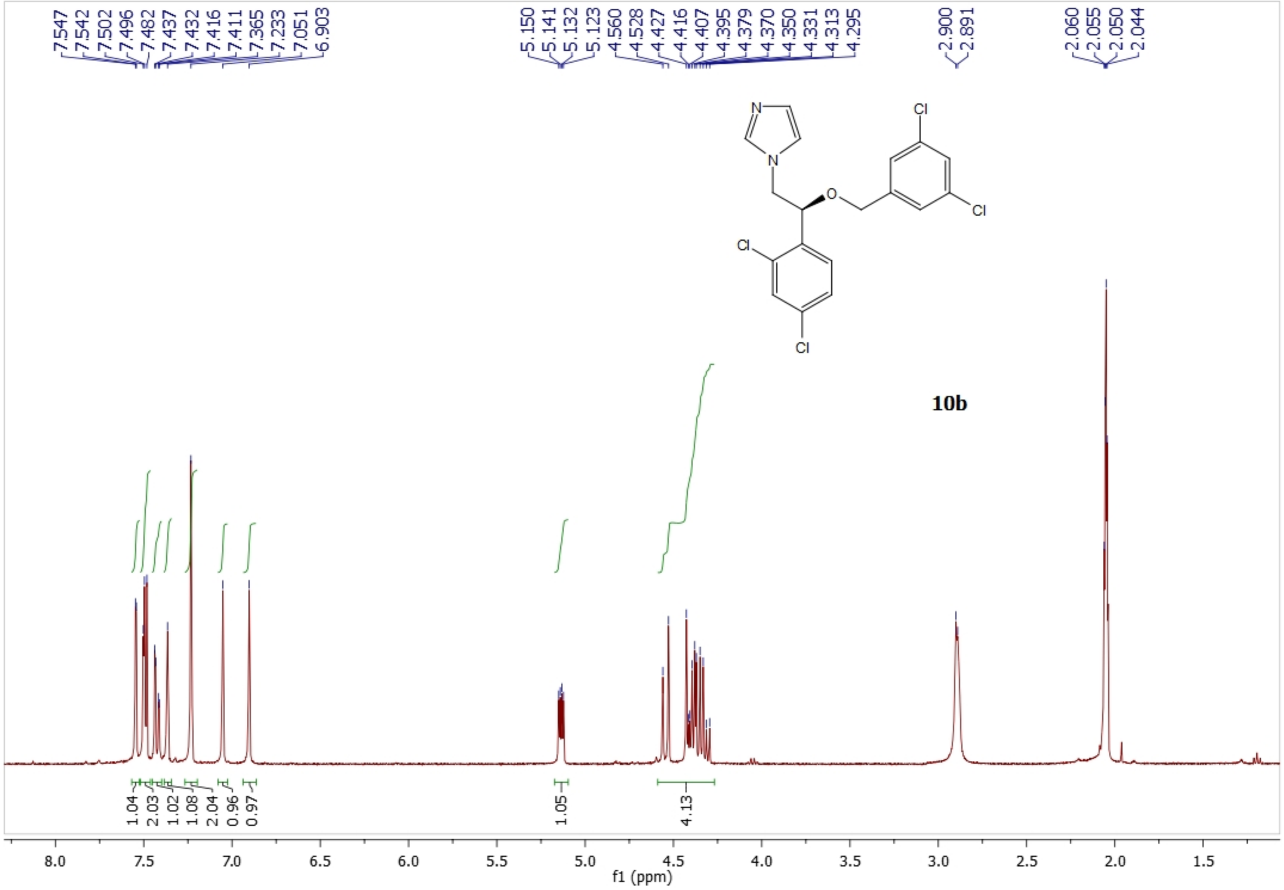
7.547
7.542
7.502
7.496
7.482
7.437
7.432
7.416
7.411
7.365
7.233
7.051
6.903
5.150
5.141
5.132
5.123
4.560
4.528
4.427
4.416
4.407
4.395
4.379
4.370
4.350
4.331
4.313
4.295
2.900
2.891
2.060
2.055
2.050
2.044
N
N
O
Cl
Cl
Cl
Cl
10b
1.04
2.03
1.02
1.08
2.04
0.96
0.97
1.05
4.13
8.0
7.5
7.0
6.5
6.0
5.5
5.0
4.5
4.0
3.5
3.0
2.5
2.0
1.5
f1 (ppm)

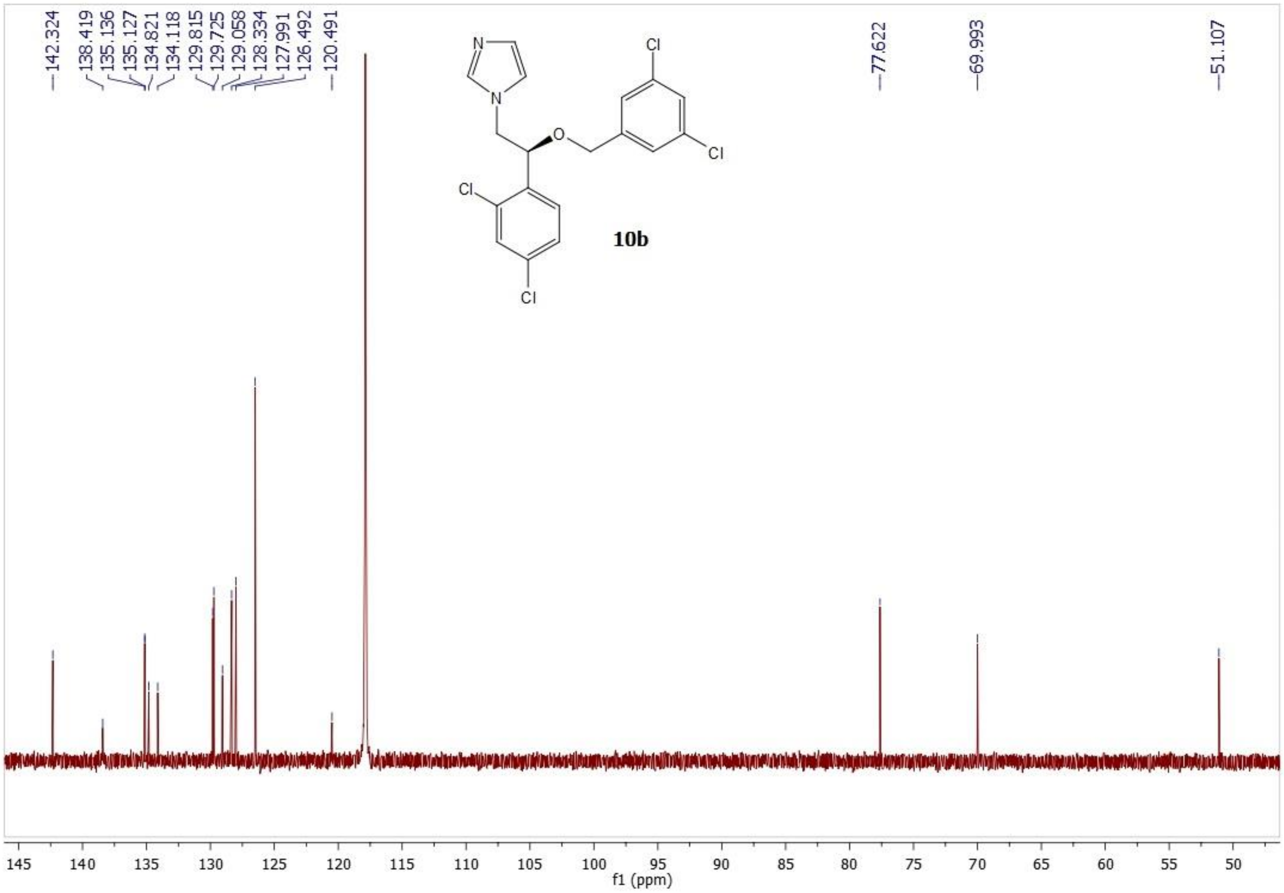
142.324
138.419
135.136
135.127
134.821
134.118
129.815
129.725
129.058
128.334
127.991
126.492
120.491
77.622
69.993
51.107
N
N
O
Cl
Cl
Cl
Cl
10b
145
140
135
130
125
120
115
110
105
100
95
90
85
80
75
70
65
60
55
50
f1 (ppm)

HPLC trace of derivative **8b**

==== Shimadzu LCsolution Analysis Report ====

C:\LabSolutions\Data\Project1\347_1.lcd

Acquired by	: Admin
Sample Name	: 347_1
Sample ID	: 347_1
Vail #	: 0
Injection Volume	: 50 uL
Data File Name	: 348_1.lcd
Method File Name	: MeOH 87 H2O 13 isocratica Podust.lcm
Batch File Name	:
Report File Name	: Default.lcr
Data Acquired	: 21/04/2022 15.00.04
Data Processed	: 21/04/2022 15.06.27

<Chromatogram>

C:\LabSolutions\Data\Project1\347_1.lcd

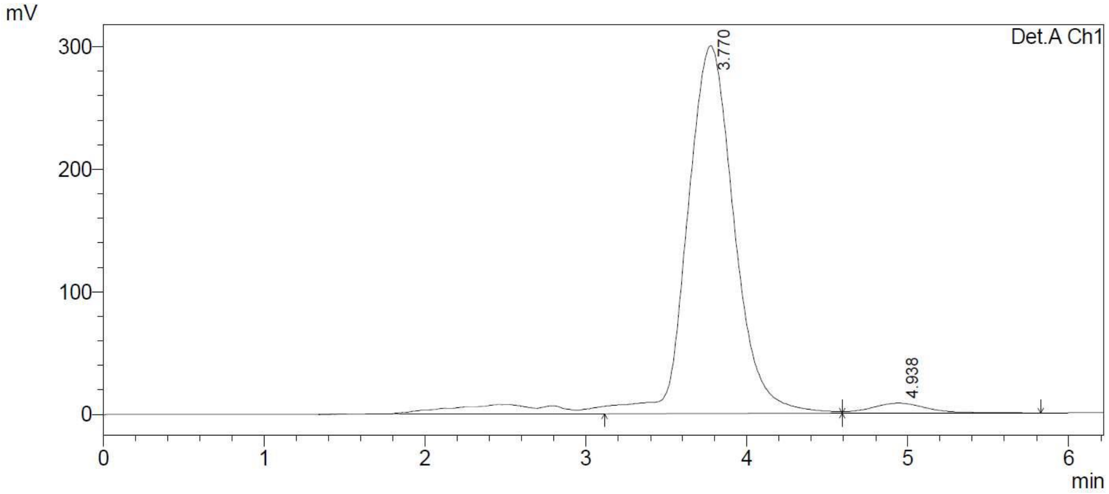

1 Det.A Ch1/254nm

PeakTable
Detector A Ch1 254nm

Peak#	Ret. Time	Area	Height	Area %	Height %
1	3.770	9583258	439023	97.54	97.73
2	4.938	241895	10213	2.46	2.27
Total		9825153	449236		

HPLC trace of derivative **9b**

==== Shimadzu LCsolution Analysis Report ====

C:\LabSolutions\Data\Project1\348_1.lcd

Acquired by : Admin
Sample Name : 348_1
Sample ID : 348_1
Vail # : 0
Injection Volume : 50 uL
Data File Name : 348_1.lcd
Method File Name : MeOH 87 H2O 13 isocratica Podust.lcm
Batch File Name :
Report File Name : Default.lcr
Data Acquired : 21/04/2022 15.12.41
Data Processed : 21/04/2022 15.18.49

<Chromatogram>

C:\LabSolutions\Data\Project1\348_1.lcd

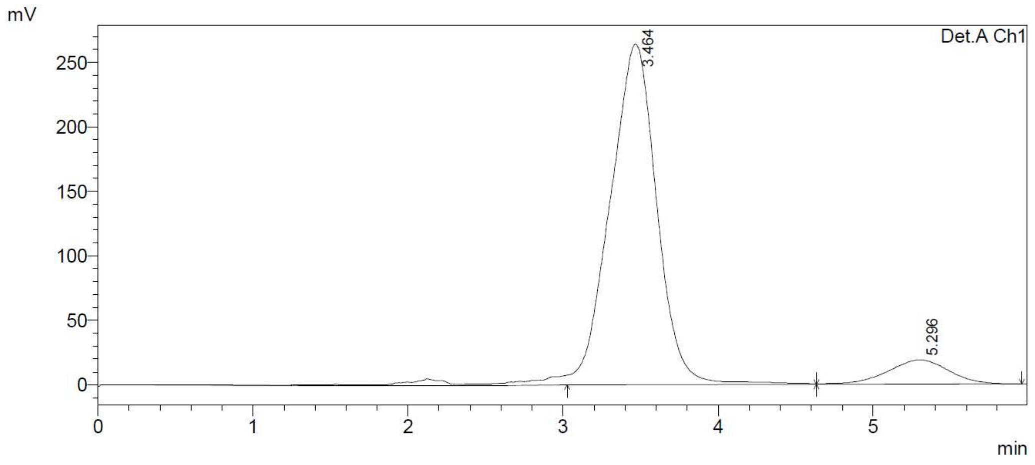

1 Det.A Ch1/254nm

PeakTable
Detector A Ch1 254nm

Peak#	Ret. Time	Area	Height	Area %	Height %
1	3.464	5493258	264897	90.72	93.348
2	5.296	561988	18813	9.28	6.652
Total		6055246	283710		
